# A Two-Stage Automatic Color Thresholding Technique

**DOI:** 10.3390/s23063361

**Published:** 2023-03-22

**Authors:** Shamna Pootheri, Daniel Ellam, Thomas Grübl, Yang Liu

**Affiliations:** 1 HP-NTU Digital Manufacturing Corporate Lab, Nanyang Technological University, Singapore 639798, Singapore; 2 HP Security Lab, HP Inc., Bristol BS1 6NP, UK

**Keywords:** image binarization, robust color thresholding, image segmentation, histogram analysis, printed circuit assembly board inspection, medical image analysis

## Abstract

Thresholding is a prerequisite for many computer vision algorithms. By suppressing the background in an image, one can remove unnecessary information and shift one’s focus to the object of inspection. We propose a two-stage histogram-based background suppression technique based on the chromaticity of the image pixels. The method is unsupervised, fully automated, and does not need any training or ground-truth data. The performance of the proposed method was evaluated using a printed circuit assembly (PCA) board dataset and the University of Waterloo skin cancer dataset. Accurately performing background suppression in PCA boards facilitates the inspection of digital images with small objects of interest, such as text or microcontrollers on a PCA board. The segmentation of skin cancer lesions will help doctors to automate skin cancer detection. The results showed a clear and robust background–foreground separation across various sample images under different camera or lighting conditions, which the naked implementation of existing state-of-the-art thresholding methods could not achieve.

## 1. Introduction

Thresholding is a key computer vision (CV) technique for image segmentation. It is an important image pre-processing step for many applications, such as medical image analysis [1,2], satellite image analysis [3], the spatio-temporal analysis of videos [4,5], and text document analysis [6,7]. Thresholding helps to distinguish the foreground or region of interest (ROI) from the background by grouping pixels into distinct sets based on features such as grey-level changes, edges, texture, color, smoothness, and pixel connectivity [8]. Thresholding techniques can be broadly categorized into global and local methods. Global thresholding applies a fixed threshold value to the entire image in order to separate background and foreground pixels. The local thresholding approach adjusts the threshold value to different subregions in the image and is therefore dependent on the subregion. Concretely, local thresholding algorithms determine the threshold based on the pixel values around a small region or block [9]. As a result, better segmentation within subregions can be achieved. However, recalculating the threshold for each subregion in an image can be computationally expensive. Furthermore, choosing an optimal block size for local thresholding is critical, since the wrong block size may cause blocking artefacts and result in poor foreground–background separation [10]. Issues arise when natural variations occur within an image dataset caused by artefacts such as varying background colors, lighting differences, and camera specifications, to name a few. A thresholding system resilient to these natural and often-occurring deviations in real-life applications is needed to automate the thresholding process, and existing local and global techniques are insufficient to address these requirements.

Conventional thresholding techniques segment the image into two or more classes based on the grayscale value, which holds the intensity information of a pixel. Building upon grayscale thresholding, traditional color thresholding methods use the color intensity to determine a threshold value [8]. Well-established, commonly used methods for grayscale thresholding include Otsu’s [11] and Kapur’s [12] methods, which were initially proposed for grayscale images and later tailored for RGB-based color images thresholding [1,13,14,15,16,17]. RGB-based color thresholding techniques separate the foreground and background pixels based on the gray value of each RGB channel. In addition, in RGB-based image representation, the intensity of the image pixels is not decoupled from the chromaticity, and there is a high correlation between the R, G, and B channels. Thus, RGB channel-based color thresholding techniques are susceptible to intensity changes and are not reliable for thresholding color images. Therefore, other color spaces are generally applied to image processing, such as the HSV (hue, saturation, value) space or the CIE L*a*b* space, where the chromatic features are decoupled from the intensity parameters [8]. In this paper, we propose a two-stage global–local HSV-based color thresholding technique. The main contributions of our method can be summarized as follows:

1. A fully automated histogram-based color thresholding approach is provided, which is invariant to natural variations in images such as varying background colors, lighting differences, and camera specifications.
2. Block size determination and addressing the blocking artefacts problem during the local thresholding stage are achieved by automatically detecting the blocks from the global thresholded image.
3. The method represents an unsupervised technique, as it does not require any labeled data for training, making it advantageous in situations where labeled data are limited or difficult/costly to generate.

An automatic thresholding technique that adapts to image variations can provide immense value to various CV applications. Although the method is illustrated herein with the use case of thresholding images depicting printed circuit assembly (PCA) boards and skin lesion images, the techniques apply to other datasets. To motivate the techniques’ application, consider how PCA boards are becoming increasingly complex and densely packed with small components, making the clean separation of foreground and background pixels increasingly challenging. Performing this accurately makes a big difference in the ability to automate the visual inspection of PCA boards and to facilitate counterfeit or security analyses [18]. By automating the segmentation of lesions from medical images, physicians will be able to detect abnormalities clearly and make more accurate diagnoses.

The paper is organized as follows: Section 2 briefly describes fundamental image thresholding research. Section 3 presents the proposed two-stage automatic global–local color thresholding technique. Section 4 presents the implementation details, Section 5 provides details of the evaluation metrics, and Section 6 explains the experimental results. Section 7 discusses the results, and Section 8 describes the application areas. Section 9 presents the limitations and future directions of the proposed method, and Section 10 concludes the paper.

## 2. Related Work

In digital image processing, thresholding is a well-known technique for image segmentation. Because of its wide applicability, a range of different thresholding methods have been proposed over the years [9,19]. Histogram-based image segmentation methods are one of the most promising conventional computer vision techniques to separate foreground objects from an image background, and various types are presented in the literature [8,9,12,20,21,22,23]. Otsu’s method automatically finds an optimal threshold value from the intensity of the image histogram, which minimizes intra-class intensity variation between the foreground and background classes [24]. Bhandari [3] and Kapur’s [12] methods find the optimal threshold value by minimizing the cross-entropy between the original image and the thresholded image using the histogram. They have been used for decades in combination with other techniques. As an example, Su et al. [25] and Qi et al. [26] recently proposed multilevel thresholding methods that apply 2D histograms, 2D Kapur entropy, and non-local means to segment chest X-ray images. Otsu’s intraclass variation method and Kapur’s entropy-based techniques are promising for images with bimodal histograms [8], but they are not suitable for images with multimodal or unimodal histograms that have small foreground regions such as the PCA boards. Parker [27] described p-tile, two-peak, and local contrast thresholding. The p-tile method is one of the oldest basic histogram-based thresholding methods, which requires the manual input of a desired black or white pixel ratio. The two-peak method locates the valley between two peaks in a gray-level histogram and defines the valley as the ideal threshold value. Local contrast thresholding segments an image by enhancing the edges and then classifying the pixels based on the contrast measure of the gray-level co-occurrence matrix. Niblack [23] introduced a variable histogram-based thresholding method where the threshold value is dynamically adjusted to the mean and standard deviation in a neighborhood of a particular pixel. Sauvola et al. [28] proposed a local adaptive thresholding method that uses soft decision-based thresholding for non-textual components and histogram-based thresholding for textual components. To partition the image into textual and non-textual regions, they computed the average gray values and the transient difference of local windows.

Histogram concavity analysis methods have been employed for decades. Early research in this field [2,29,30] has shown that selecting thresholds based on the valleys or shoulders (concavities) of a histogram function leads to the sufficient separation of background and foreground pixels in an image. A fundamental histogram concavity analysis technique is the mode method, which was applied by Prewitt and Mendelsohn [2] to analyze images of human blood cells. This method places thresholds at the local minima of a smoothened histogram. To further isolate significant peaks and valleys from insignificant ones, several early methods have been proposed, such as weighting pixels according to the values of neighboring pixels [31] or recursively thresholding the histogram function [32]. Compared to cluster-based methods such as the p-tile method [33], which segments the image based on a manually pre-defined object area, histogram concavity methods have the advantage of automatically grouping pixels based on the result of the concavity analysis and therefore reducing the likelihood of inadvertently classifying foreground objects as background. However, one limitation of concavity analysis, pointed out by [9], is the lack of resilience when it comes to histograms with sharp peaks and elongated shoulders.

Text document binarization is an important task in the field of document analysis and image processing. Over the years, many techniques have been proposed in the literature for text document binarization [34,35]. Wolf and Jolion [36] proposed a text segmentation system that encompasses a contrast-based binarization technique combining the segmentation quality of Niblack’s method and the robustness of Sauvola’s method. Feng and Tan [37] described a local thresholding method for binarizing low-quality text images based on the gray values of two local windows. They compared the gray-value standard deviations of the primary (smaller) and secondary (larger) local window to separate text regions from background regions. Another well-suited method for text segmentation was introduced by Shaikh et al. [7], who used an iterative partitioning approach that calculates an optimal grayscale threshold value for different cells in an image. It works particularly well on text document images with a noisy background. Bradley and Roth’s [5] method thresholds pixels based on the average pixel value of a surrounding square window of pixels. To compute the averages, they used the integral image to accomplish the computation in linear time. Singh et al. [38] also used a local contrast thresholding technique based on the mean pixel value, minimum, and maximum of a local window for binarizing noisy and text document images. Sukesh et al. evaluated various deep-learning-based text document binarization techniques in [39].

Medical image thresholding is a critical task in medical image processing and analysis that involves segmenting an image into distinct regions based on intensity values [40]. Feng et al. [41] used a multi-scale 3D Otsu thresholding algorithm for medical image segmentation. Fazilov et al. [42] incorporated Otsu’s method for mammographic image segmentation. Kapur’s thresholding technique was used in [43] for detecting tumors in MRI images using a transformed differential evolution algorithm. One of the most widely used network and training strategies for biomedical image segmentation is U-Net [44,45]. It works with few training images and outputs more precise segmentations than its predecessors. Venugopal et al. [46] proposed DTP-Net, a deep convolutional neural network (DCNN) that aims to predict the optimal grayscale threshold value for an image. Their method is tailored to binarizing lesions on dermatological macro-images. Similarly, Han et al. [47] described a DCNN-based skin lesion image segmentation method. They trained their HWA-SegNet model with the image’s 2D discrete Fourier transform frequency information and further fine-tuned the edge information of skin lesions. There has also been an increase in newly published image segmentation methods that are specifically tailored to thresholding magnetic resonance and X-ray images. Chen et al. [48] introduced a transformer-based method that incorporates multilevel region and edge information and thus achieved high DSI scores on their magnetic resonance image test dataset. To further improve the run-time quality and segmentation performance of such methods, Uslu and Bharath [49] proposed a quality control method that ultimately aims to increase the trustworthiness of DCNN-based methods in the medical image analysis field.

Many techniques [50,51,52] in the recent literature do not analyze the chromaticity information to threshold color images, since they incorporate RGB-channel-based thresholding. While some [10,53,54] do apply HSV or L*A*B* color space analysis to thresholding problems, they do not automatically determine the threshold limits based on the unique characteristics of the image. Our proposed method determines the chromaticity of the background or foreground pixels using the hue and saturation histograms and computes the optimal color threshold by considering the changes in histogram gradient and histogram cumulative area. It is suitable for thresholding images with unimodal, bimodal, and multimodal histograms and histograms with sharp peaks or elongated shoulders compared to other histogram-based thresholding techniques. Thresholding solely based on histogram valleys and shoulders has a significant disadvantage compared to our proposed method. Depending on the characteristics of a valley, a suboptimal threshold might be computed. Moreover, histogram concavity analysis (valley and shoulder analysis) is not suitable for thresholding images with bimodal or multimodal histograms.There are advanced computer vision techniques using DCNN aimed at developing medical image segmentation approaches [46,55]. The DCNN-based methods do not typically work well when thresholding small objects [56] such as PCA board components. In addition, DCNN models require a large set of labeled training data, which is not feasible as it requires the manual labeling of small PCA board components.

In this paper, we propose a two-stage histogram-based automatic color thresholding technique based on the chromaticity of the image pixels. The proposed method is an unsupervised technique, as it does not require any labeled data like deep learning-based thresholding techniques [46,55]. The details of the proposed method are provided in the following section.

## 3. Methods

**Figure 1:**
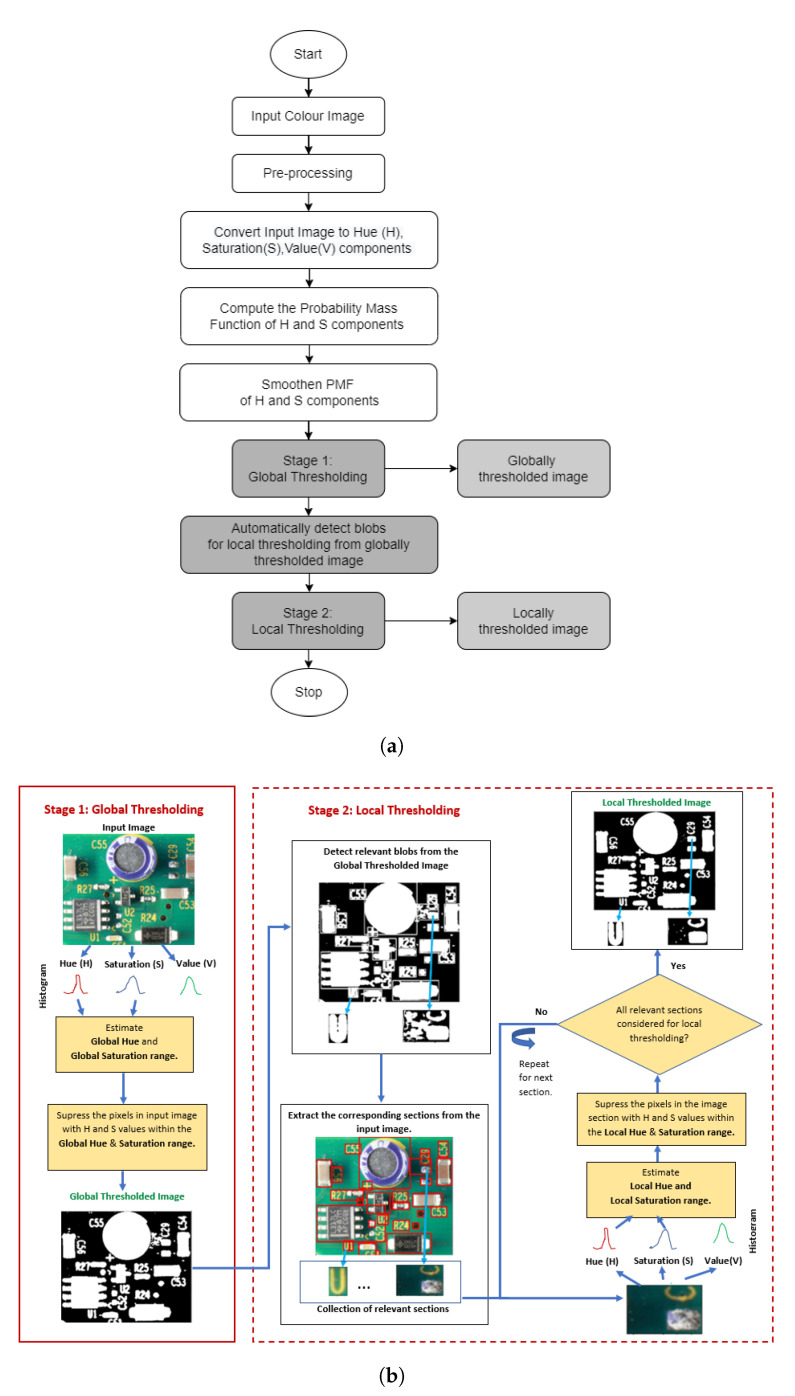
(**a**) Overall sequence of the two-stage global–local thresholding technique. Gray-shaded boxes indicate the key contributions of the proposed method. (**b**) Schematic overview of the global–local thresholding stages.

In this section, we explain the proposed two-stage histogram-based automatic color thresholding technique to segment an image into foreground and background regions. Figure 1 provides a high-level overview of the entire technique, and Algorithm 1 describes the global and local thresholding stages. Initially, the image is converted to the hue (H), saturation (S), and value (V) format. In the HSV format, the chromaticity components (H and S) are decoupled from the intensity component (V). The intensity component V is excluded from the threshold computation to avoid illumination changes.

**Algorithm 1** Two-stage global–local thresholding
**Input:**


Img

▹ Input color image

Cutoff_Gradient

▹ Threshold value for histogram Avg_Gradient

Cutoff_Area

▹ Threshold value for histogram Avg_Area

Window_Size

▹ Window size for calculating the Avg_Gradient and Avg_Area

Limit1

▹ The limit for maximum continuous hue range

Limit2

▹ The limit for maximum continuous saturation range

C1

▹ A constant that determines the degree of change from global hue to local hue

C2

▹ A constant that determines the degree of change from global saturation to local saturation
**Output:**


GT_Img

▹ Globally thresholded image, background in black and foreground in white

LT_Img

▹ Locally thresholded image, background in black and foreground in white
**Stage 1: Global Thresholding **  1:Let H, S, and V be the hue, saturation, and value components of Img2:

PMFH←compute_pmf(H)

3:

Smoothened_PMFH←smoothening(PMFH)

4:PeakH←argmax(Smoothened_PMFH)     ▹ Index of max H component from Smoothened_PMF5:

Nominated_RangeGH←nominated_range(Smoothened_PMFH)

6:

Result_GH←{}

7:**while** PeakH not in Result_GH **do**    ▹ Ensures that the global nominated hue range includes the PeakH8:    Nominated_Range_GH←Nominated_Range_GH−Result_GH9:    Result_GH←max_continuous_range(Nominated_Range_GH,Limit1)10:
**end while**
11:

Global_H_Range(GH_low,GH_high)←[min(Result_GH),max(Result_GH)]

12:

Shortlisted_S←find_sat_values(GH_low,GH_high,H,S)

13:

PMFS←compute_pmf(Shortlisted_S)

14:

Smoothened_PMFS←smoothening(PMFS)

15:

Nominated_Range_GS←nominated_range(Smoothened_PMFS)

16:

Result_GS←max_continuous_range(Nominated_Range_GS,Limit2)

17:

Global_S_Range(GS_low,GS_high)←[min(Result_GS),max(Result_GS)]

18:

GT_Img←threshold(Img,Global_H_Range(GH_low,GH_high),Global_S_Range(GS_low,GS_high))

19:

LT_Img←local_threshold(Img,GT_Img,Global_H_Range(GH_low,GH_high),Global_S_Range(GS_low,GS_high))

**Stage 2: Local Thresholding**
  20:**function** local_threshold(Img,GT_Img,Global_H_Range(GH_low,GH_high),Global_S_Range(GS_low,GS_high))21:    Final_Image←zeros(Img.width,Img.height)   ▹ Initialize a blank image with the same size as the input image Img22:    Blobs←blobs in GT_Img          ▹ Detect all blobs from GT_Img23:    Relevant_blobs←select_relevant_blobs(Blobs,minimum_Size=500)   ▹ Blobs with area ≥ 500 pixels are considered relevant24:    **for** *b* in Relevant_blobs **do**25:        Let [(min_x,min_y),(max_x,max_y)] be the location coordinate of blob ’b’ in GT_Img26:        Cropped←Img[min_x:max_x,min_y:max_y]   ▹ Crops corresponding section from the input image Img27:        H_local,S_local, and V_local← hue, saturation, and value components of Cropped section28:        H_local_confined←H_local[(GH_low−C1):(GH_high+C1)]  ▹ Confines local hue to global hue range ± C1 to eliminate the hues of foreground components in the local region29:        PMFH_Local←compute_pmf(H_local_confined)30:        Smoothened_PMFH_Local←smoothening(PMFH_Local)31:        Nominated_Range_LH←nominated_range(Smoothened_PMFH_Local)32:        Result_LH←max_continuous_range(Nominated_Range_LH,Limit2)33:        Local_H_Range(LH_low,LH_high)←[min(Result_LH),max(Result_LH)]34:        Shortlisted_S←find_sat_values(Local_H_Range(LH_low,LH_high),H_local,S_local)35:        S_local_confined←Shortlisted_S[(GS_low−C2):(GS_high+C2)]  ▹ Confines local saturation to global saturation range ± C2, to eliminate the saturation of foreground components in the local region36:        PMFS_Local←compute_pmf(S_local_confined)37:        Smoothened_PMFS_Local←smoothening(PMFS_Local)38:        Nominated_Range_LS←nominated_range(Smoothened_PMFS_Local)39:        Result_LS←max_continuous_range(Nominated_Range_LS,Limit2)40:        Local_S_Range(LS_low,LS_high)←[min(Result_LS),max(Result_LS)]41:        Cropped_Thresholded←threshold(Cropped,Local_H_Range(LH_low,LH_high),Local_S_Range(LS_low,LS_high))42:        Final_Image[min_x:max_x+1,min_y:max_y+1]←Cropped_Thresholded43:    **end for**44:    **return** Final_Image45:
**end function**


**Helper Functions**  46:**function** smoothening(histogram,window_size←5)47:    smoothened_histogram←zeros(len(histogram))48:    **for** *i* in range(len(histogram)) **do**49:        lower_bound←max(0,i−window_size/2)50:        upper_bound←min(len(histogram),i+window_size/2)51:        window_sum←052:        **for** *j* in range(lower_bound,upper_bound) **do**53:           window_sum←window_sum+histogram[j]54:           smoothened_histogram[i]←window_sum/((upper_bound−lower_bound)+1)55:        **end for**56:    **end for**57:    **return** smoothened_histogram58:
**end function**
59:**function** compute_pmf(Hist)60:    N←sum(Hist)61:    pmf←zeros(len(Hist))62:    **for** *i* in range(len(Hist)) **do**63:        count←Hist[i]64:        pmf[i]←count/N65:    **end for**66:    **return** pmf67:
**end function**
68: 69:**function** nominated_range(Smoothened_PMF,window_size←5)70:    Nominated_Range←{}71:    **for each** *h* in Smoothened_PMF **do**72:        **if** (*h* is not in Nominated_Range) **then**   ▹ Compute the Avg_Gradient and Avg_Area within the window73:           h_lower←max(0,h−window_size/2)74:           h_upper←min(len(Smoothened_PMF),h+window_size/2)75:           $Avg\_Area\leftarrow \frac{\sum_{i=h\_lower}^{h\_upper}Smoothened\_PMF\left(i\right)}{(h\_upper-h\_lower)+1}$76:           **if** (h_upper<180) **then**77:               $Avg\_Gradient\leftarrow \frac{\sum_{i=h\_lower}^{h\_upper}\arctan(|Smoothened\_PMF\left(i\right)-Smoothened\_PMF\left(i+1\right)|)}{(h\_upper-h\_lower)+1}$78:           **else**79:               $Avg\_Gradient\leftarrow \frac{\sum_{i=h\_lower}^{h\_upper-1}\arctan(|Smoothened\_PMF\left(i\right)-Smoothened\_PMF\left(i+1\right)|)}{\left(h\_upper-h\_lower\right)}$80:           **end if**81:           **if** (Avg_Gradient≥Cutoff_Gradient) **or** (Avg_Area≥Cutoff_Area) **then**82:               Nominated_Range←Nominated_Range∪{h_lowertoh_upper}83:           **end if**84:        **end if**85:    **end for**86:    **return** Nominated_Range87:
**end function**
88: 89:**function** max_continuous_range(range_input,Limit)90:    result←{}91:    current←{}92:    **for** *r* in range_input **do**93:        **if** (len(current)==0)**or** (|r−current[−1]|<=Limit) **then**94:           current←current∪{r}95:        **else**96:           **if** (len(current)>len(result)) **then**97:               result←current98:           **end if**99:        **end if**100:        current←{r}101:    **end for**102:    **if** (len(current)>len(result)) **then**103:        result←current104:    **end if**105:    **return** result106:
**end function**
107: 108:**function** find_sat_values(H_low,H_high,H,S)109:    Sat←{}110:    **for** *i* in range(len(H)) **do**111:        **if** (H_low≤H[i]≤H_high) **then**112:           Sat←Sat∪{S[i]}113:        **end if**114:    **end for**115:    **return** Sat116:
**end function**
117:**function** threshold(image,H_Range(H_low,H_high),S_Range(S_low,S_high))118:    Thresholded_image←zeros(image.height,image.width)119:    **for** *i* in range(image.height) **do**120:        **for** *j* in range(image.width) **do**121:           H← hue component of image[i][j]122:           S← saturation component of image[i][j]123:           **if** ((H_low≤H≤H_high) **and** (S_low≤S≤S_high)) **then**124:               Thresholded_image[i][j]←0125:           **else**126:               Thresholded_image[i][j]←255127:           **end if**128:        **end for**129:    **end for**130:    **return** Thresholded_image131:
**end function**
132: 133:**function** select_relevant_blobs(blobs,minimal_size)134:    relevant_blobs←{}135:    **for** blob in blobs **do**136:        area←height(blob)×width(blob)137:        **if** (area≥minimal_size) **then**138:           relevant_blobs←relevant_blobs∪{blob}139:        **end if**140:    **end for**141:    **return** relevant_blobs142:
**end function**



After splitting the image into HSV components, a hue probability mass function (PMF) is computed from the hue histogram component of the input image. In order to reduce unwanted noise, the PMF is smoothened. It is computationally advantageous to perform image smoothening on the image’s histogram rather than on the image itself, which effectively binds all necessary computations to a fixed-size histogram, thus avoiding an increase in computational complexity with a growing image resolution. After smoothening the PMF, a two-stage thresholding technique is applied based on the chromaticity components to both global and local image regions. In both stages, the proposed method initially determines the probable background hue or sat values (nominated hue or sat values), and then finalizes the optimal background hue or sat-range selection (max continuous hue or sat range) from the nominated values. A detailed explanation of global and local thresholding stages is provided in Section 3.1 and Section 3.2.

### 3.1. Stage 1: Global Thresholding

**Figure 2:**
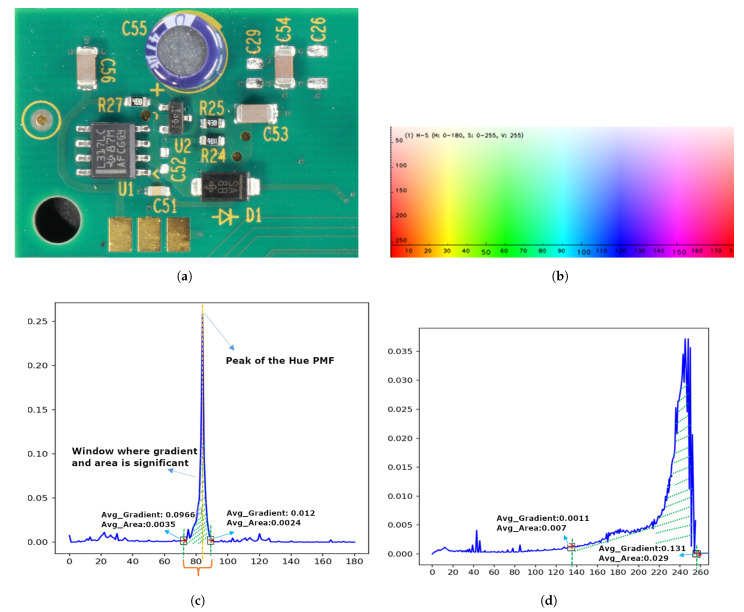
(**a**) Input color image. (**b**) Hue and saturation representation for the HSV format in OpenCV. The hue ranged from 0 to 180, the saturation ranged from 0 to 255, and the value was fixed at 255 for this representation. (**c**) The probability mass function of the hue component (symmetric unimodal histogram) of the input image. The nominated hue ranges were 18 to 35, 72 to 93, and 116 to 130, where the area under the curve and gradient were greater than a cut-off value within a window. The shaded region (72 to 93) shows the optimal global hue range selected by the proposed algorithm according to the maximum continuous hue range heuristic, including the peak hue at position 83. (**d**) The probability mass function of the saturation component of the input image (skewed unimodal histogram). The nominated saturation ranges were 30 to 55, 75 to 90, and 137 to 255, where the area under the curve and gradient were greater than a cut-off value within a window. The shaded region (137 to 255) shows the optimal global saturation range selected by the maximum continuous saturation range heuristic. The small black box indicates the window, and the red arrow represents the gradient value in the corresponding window.

Stage 1 of Algorithm 1 defines the global hue and saturation threshold ranges that apply to the entire image. Initially, the image is converted to HSV format (step 1), and the PMF of the hue component is smoothened (steps 2 and 3). A specific hue ‘h’ qualifies for the nominated hue when either the average area within a window is greater than a predefined cut-off value (Cutoff_Area) or the average slope surrounding ’h’ (a sudden change in PMF within a window) is greater than a predefined cut-off value (Cutoff_Gradient) (step 5). We applied the max continuous hue range heuristic to the nominated hue values. The global max continuous hue range is the largest range of hues, including the peak hue value (PeakH) within the nominated hue values, for which the difference between the consecutive hue values is less than a certain small threshold Limit 1 (e.g., 2) (steps 6–11). For example, as presented in Figure 2c, the nominated hue for the input PCA board image (Figure 2a) is between 18 and 130. From the hue and saturation shades shown in Figure 2b, it is evident that this range includes shades of green, yellow, and blue. From the input image presented in Figure 2a, one can infer that the hue of the yellow text and the blue capacitor caused small peaks in the PMF, and these local maxima are also included in the nominated hue values. To eliminate these foreground hues, we used the maximum continuous hue range heuristic. This estimated continuous hue range is defined as the global hue range Global_H_Range(GH_low, GH_high). We refer to this as ’global’ since this hue range is derived from all image pixels. It is applied to the whole image for thresholding; hence, it is the global hue range. In Figure 2c, the green shaded region between 72 and 93 is the global hue range, which represents the background hues of the input PCA board image presented in Figure 2a.

As depicted in step 12 of Algorithm 1, to find the global saturation range, the pixels within the global hue range are shortlisted, and the S components of the shortlisted pixels (Shortlisted_S) are collected. From the smoothened_PMF of Shortlisted_S, Algorithm 1 estimates the nominated saturation values as described in step 15. Then, we applied the maximum continuous saturation range heuristic to the nominated saturation values to obtain the global saturation range (GS_low to GS_high) (steps 16–17). The max continuous saturation range is the largest range of saturation values within the nominated saturation values, for which the difference between the consecutive saturation values is less than a certain small threshold Limit 2 (e.g., 4). The max continuous hue range heuristic must select a range including the peak hue. However, in the max continuous saturation hue heuristic, it is not compulsory to select the range including the peak saturation; the selection can also be a significant range that may/may not include the global maximum of the saturation histogram. Once the global hue and saturation ranges are fixed, the image is segmented into background and foreground regions. If a pixel’s hue is within the global hue range and its saturation is within the global saturation range, that pixel is considered a background pixel; otherwise, it is considered a foreground pixel. A globally thresholded binary image is generated by setting the intensity values of all background pixels to ‘0’ and the intensity of the foreground pixels to ’255’ (step 18). The input color image, global thresholded image, and global hue–saturation threshold ranges are passed to stage 2 (local thresholding) to further improve the results within subregions (step 19).

Figure 2 explains the estimation of the nominated and max continuous hue and saturation ranges for a given input image. As per the proposed algorithm, a specific hue ‘h’ qualifies as a nominated hue value when either the average area or the average gradient within a window (including ‘h’) is greater than a predefined Cutoff_Area or Cutoff_Gradient, respectively. We set the Cutoff_Gradient value to 0.001, the Cutoff_Area to 1/180 (1 over the length of the histogram ≈ 0.0055), and the Window_Size constant to 5 (these values were heuristically determined, and more details about the parameter settings can be found in Section 4). In Figure 2c, the average gradient (Avg_Gradient) and average area (Avg_Area) within the window (where hue 72 is the starting point of the window) are 0.0966 and 0.0035, respectively. The Avg_Gradient is greater than the Cutoff_Gradient (0.0966≥0.001), and the Avg_Area is smaller than the respective cut-off value (0.0035≤0.0055). As described earlier, either the gradient OR the area must be greater than its respective cut-off value to be considered as the nominated hue. Both the Avg_Area and the Avg_Gradient of the preceding window of hue point 72 are less than their respective cut-off values. Likewise, the Avg_Area and Avg_Gradient of the window after hue point 93 are less than the cut-off values. Hence, 72–93 are included in the nominated hue range set. Similarly, hue ranges 18 to 35 and 116 to 130 are included in the nominated set. Finally, the shaded region 72 to 93 is selected as the optimal hue range by the maximum continuous hue range heuristic. The same reasoning applies to the saturation histogram in Figure 2d. Both the Avg_Area and the Avg_Gradient of the window before saturation point 137 and after saturation point 255 are less than the cut-off values. Hence, 137–255 are included in the nominated saturation range set. Similarly, the saturation ranges 30 to 55 and 75 to 90 are also included in the nominated set. Finally, the shaded region 137–255 is selected as the optimal saturation range by the maximum continuous saturation range heuristic.

### 3.2. Stage 2: Local Thresholding

**Figure 3:**
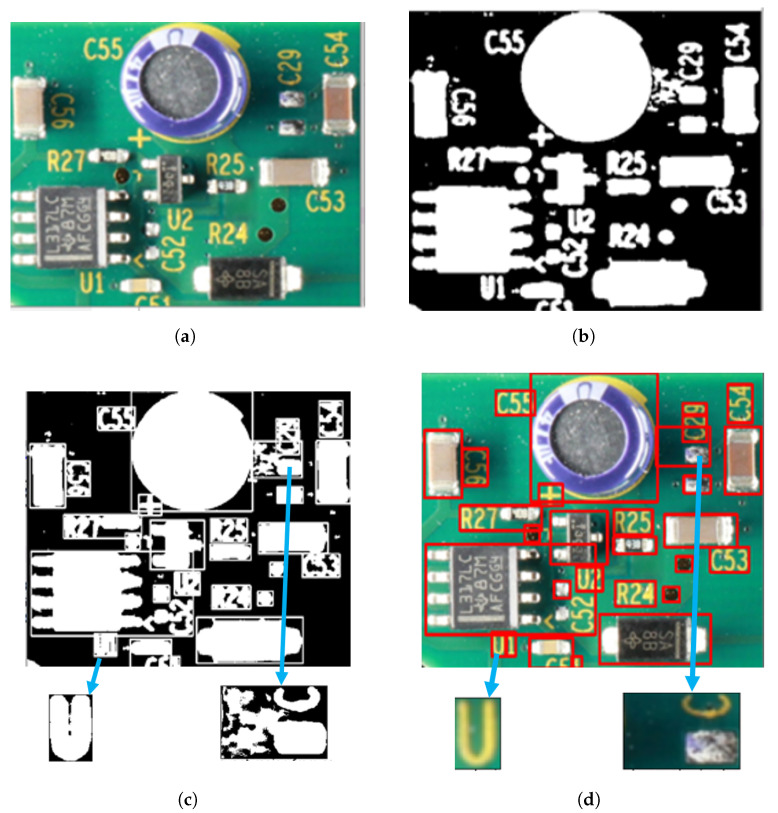

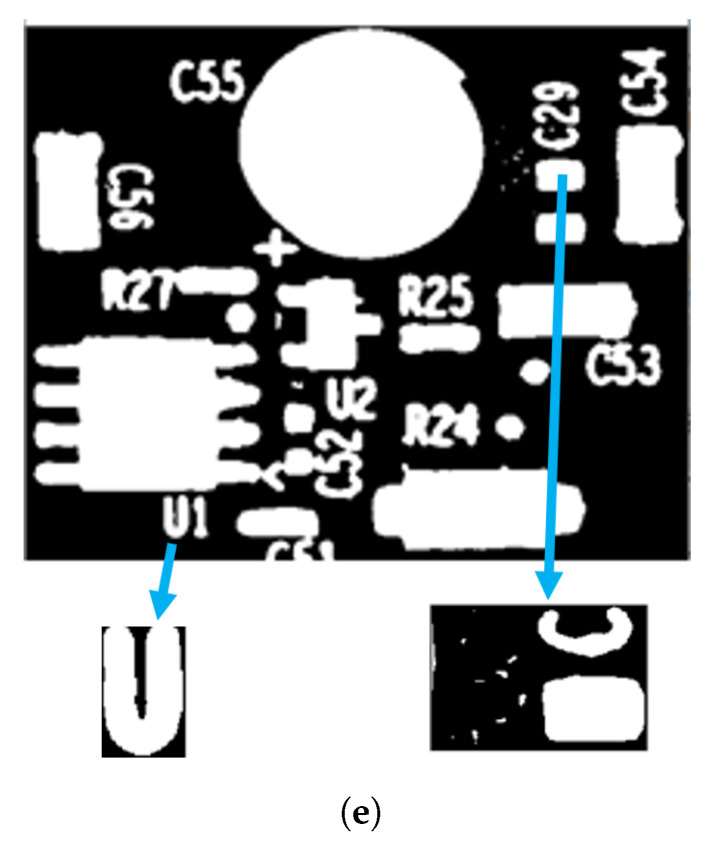
Automatic detection of local windows to refine the background. (**a**) Input color image. (**b**) Global thresholded image. Global hue range: (75, 90). Global saturation range: (111, 255). (**c**) Relevant blobs and selected regions before applying local thresholding (**d**). Relevant corresponding image regions. (**e**) Refined background after global and local thresholding. Local hue and saturation ranges of the two selected subregions: (75, 80), (102, 234) and (70, 95), (101, 252), respectively.

The second stage dynamically determines the varying local hue and saturation threshold ranges to refine the background and foreground segmentation within subregions. The locally relevant blocks or regions are detected from the global thresholded binary image (steps 22 and 23), and the detected blocks are improved using local hue and saturation thresholds. The size for local regions is automatically detected using blob detection techniques from the globally thresholded image (step 22). Areas around the blobs are extracted using a bounding box (see Figure 3c,d). We selected the relevant regions by eliminating backgrounds (areas outside the bounding boxes) and irrelevant regions (bounding boxes less than a minimal size) (step 23) for further refining. Subsequently, we picked a region from the relevant list, cropped the image section from the input color image, and computed the corresponding H, S, and V components (steps 24–27). The local H component is confined within the global hue range (step 28). This step helps to eliminate the foreground object’s hues while fine-tuning the background in the local regions. From the smoothened PMF of the local H components, the Local_H_Range(LH_low, LH_high) is computed (steps 31–33). Similarly, the local saturation range Local_S_Range(LS_low, LS_high) is computed as shown in steps 34 to 40. Thresholding is applied dynamically to the image regions based on the corresponding local hue and saturation ranges, and anything within the threshold range is classified as background (steps 41 and 42).

## 4. Implementation Details

This section presents the implementation details and parameter settings for the proposed algorithm. For our implementation, we used Python 3.10 and the OpenCV image processing library [57]. The main parameters for Algorithm 1 are Cutoff_Gradient, Cutoff_Area, Window_Size, Limit1, Limit2, C1, and C2. These values are determined heuristically, and the following section explains how we fine-tuned these parameters to obtain optimal results.

A specific hue ‘h’ qualifies as the nominated hue when either the average area around h (within the Window_Size) is greater than Cutoff_Area or the average slope surrounding ‘h’ (within the Window_Size) is greater than the Cutoff_Gradient. We set the Cutoff_Gradient value to 0.001 and the optimal Cutoff_Area to 1/180 (1 over the length of the histogram ≈ 0.0055). The Window_Size constant was set to 5, which effectively calculates the average gradient and area within a window of five consecutive values.

When determining the continuous histogram ranges that qualify as a potential background hue or saturation, we introduced Limit1 (hue continuity) and Limit2 (saturation continuity), which refer to the maximum number of consecutive points on the histogram x-axis that could lie outside of our desired gradient or cumulative area. These parameters provide some flexibility when it comes to color discontinuities or variations in the background. Limit1 defines the allowable hue discontinuity, and Limit2 defines the allowable saturation discontinuity. The constants C1 and C2 define the degree of change in local hue and saturation from the global values. The optimal values for these constants depend on the variance in the image background chromaticity in local regions. If there are limited changes in background chromaticity (no shadows or different shades), a smaller value is sufficient. Otherwise, a higher value is required to perform accurate thresholding on local blobs. Please refer to Appendix B for a collection of configuration examples.

## 5. Evaluation Metrics

We evaluated the performance of the image thresholding techniques using the following evaluation metrics, where GT refers to the ground-truth image and T refers to the thresholded image.

### 5.1. Dice Similarity Index

The Dice similarity index (*DSI* ), or Dice similarity coefficient (*DSC*), is commonly used in computer vision tasks to measure the spatial overlap of two images [58] and is defined in Equation (1). The *DSI* is twice the area of the overlap divided by the total number of pixels in both images. A high *DSI* score indicates a large spatial overlap between *GT* and the thresholded image *T*.

(1) $$DSI\left(GT,T\right)=\frac{2(|GT\cap T|)}{\left(|GT|+|T|\right)}$$

### 5.2. Matthews Correlation Coefficient

The Matthews correlation coefficient (*MCC*) is a more reliable evaluation metric for binary classification tasks [59]. It considers the true positives, false positives, true negatives, and false negatives:

- Correctly predicted foreground pixels are considered true positives (*TP*)—the number of pixels segmented as foreground in both *GT* and *T* images.
- Falsely predicted foreground pixels are considered false positives (*FP*)—the number of pixels segmented as foreground in *T* and background in *GT*.
- Correctly predicted background pixels are considered true negatives (*TN*)—the number of pixels segmented as background in both *GT* and *T* images.
- Falsely predicted background pixels are considered false negatives (*FN*)—the number of pixels segmented as background in *T* and foreground in *GT*.

The *MCC* calculates the Pearson product-moment correlation coefficient between the thresholded image *T* and a ground-truth image *GT* and is defined in Equation (2); the higher the *MCC* score, the higher the thresholding accuracy.

(2) $$MCC=\frac{TP\times TN-FP\times FN}{\sqrt{\left(TP+FP)\times (TP+FN)\times (TN+FP)\times (TN+FN\right)}}$$

### 5.3. Peak Signal-to-Noise Ratio

The peak signal-to-noise ratio (*PSNR*) is commonly used to evaluate the overall quality of an image. It is defined as the “proportion between maximum attainable powers and the corrupting noise that influence likeness of image” [60]. The *PSNR* is calculated as shown in Equation (3); the higher the *PSNR* value, the higher the thresholding accuracy.

(3) $$PSNR=20\times log_{10}\left(\frac{MAX_{I}}{\sqrt{MSE}}\right)$$

The $MAX_{I}$ value refers to the maximum intensity value. In our case, $MAX_{I}$ was set to 255, which is the highest possible value in an 8-bit grayscale image. The mean squared error (*MSE*) between *GT* and the thresholded image *T* is defined in Equation (4), where $GT_{ij}$ and $T_{ij}$ represent the ground-truth image and the thresholded image intensity at the ${\left(i,j\right)}^{th}$ position, respectively, and m,n are the height and width of the *GT* and *T* images.

(4) $$MSE=\frac{1}{mn}\sum_{i=0}^{m-1}\sum_{j=0}^{n-1}{\left(GT_{ij}-T_{ij}\right)}^{2}$$

## 6. Results

**Figure 4:**
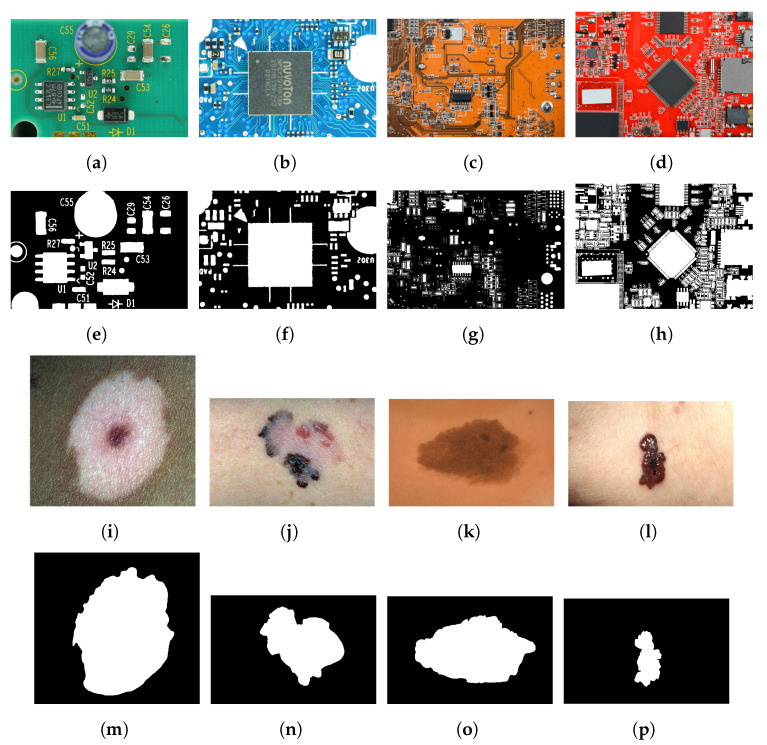
(**a**–**d**) Sample images of the PCA dataset and (**e**–**h**) corresponding ground truth. (**i**–**l**) Sample images of skin cancer dataset and (**m**–**p**) corresponding ground truth.

We evaluated the proposed method using a skin cancer image dataset and a PCA board image dataset. We tested our method on the University of Waterloo skin cancer database [61], which contains 206 images and their corresponding ground-truth images. The PCA board dataset consists of 50 images of PCA boards with varying image quality and background colors. Our team captured 44 images of PCA boards with different image resolutions and lighting conditions and blue and green board background colors. To incorporate color variability, we downloaded six PCA board images with red, yellow, and orange backgrounds from a free stock image website [62]. The ground-truth images of the 50 PCA board images were produced using a semi-automatic process: global thresholding followed by manual adjustments. Figure 4 provides sample images and corresponding ground truths of the PCA board and skin cancer image datasets. The skin cancer dataset consists of macro skin images featuring lesions with a bimodal histogram, and the PCA board image dataset consists of a background featuring different colored foreground components with unimodal or multimodal histograms. The performance of our proposed two-stage thresholding technique was evaluated quantitatively and qualitatively against other state-of-the-art thresholding techniques [5,11,12,23,27,28,36,37,38,46], and the results are presented in Section 6.1 and Section 6.2.

### 6.1. Experimental Results Using the Skin Cancer Dataset

This section provides the experimental details for state-of-the-art thresholding techniques [5,11,12,23,27,28,36,37,38,44,46] using the University of Waterloo skin cancer database [61]. The authors of the DTP-Net thresholding method [46] provided a pre-trained network that was trained with a custom dataset of 4550 skin cancer images. The custom dataset was created by merging images of melanoma and nevus lesions from the University of Waterloo skin cancer database [61] and the MED-NODE [63], SD-260 [64], and SD-198 [65] databases. Ground-truth images of all 4550 images are not publicly accessible, and only the University of Waterloo skin cancer database contained the ground-truth images for evaluation. Hence, we used the University of Waterloo skin cancer database to compare the performance of DTP-Net and all other methods shown in Table 1.

**Figure 5:**
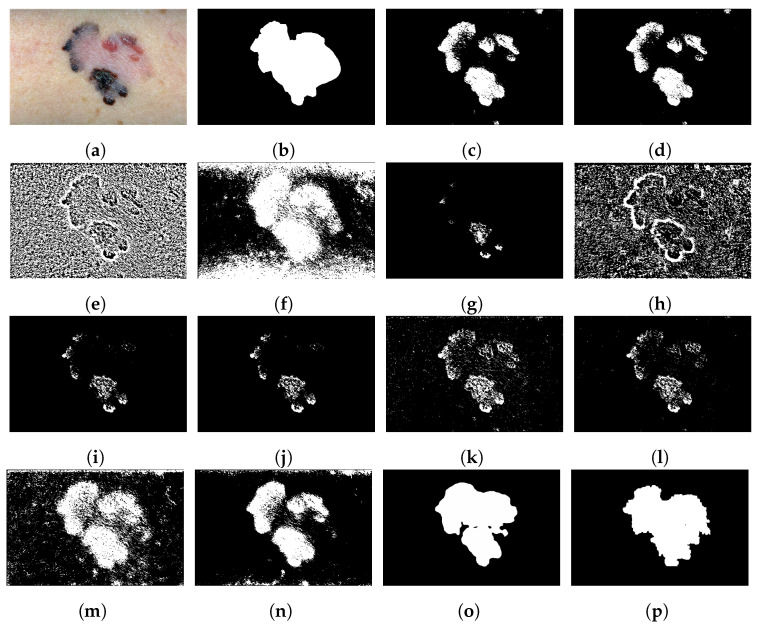
Sample thresholding results using a skin lesion image: (**a**) input image, (**b**) ground truth, (**c**) Otsu [11], (**d**) Kapur et al. [12], (**e**) Niblack [23], (**f**) P-tile [27], (**g**) two-peak [27], (**h**) local contrast [27], (**i**) Sauvola et al. [28], (**j**) Wolf and Jolion [36], (**k**) Feng and Tan [37], (**l**) Bradley and Roth [5], (**m**) Singh et al. [38], (**n**) DTP-NET [46] pre-trained model, (**o**) U-Net [44] with Resnet-152 as backbone, (**p**) proposed method.

The DTP-Net [46] performance was evaluated by fine-tuning the pre-trained model and training the model from scratch in addition to testing the pre-trained model provided by the authors. We evaluated U-Net [44] with the Resnet-152 [66] architecture as the backbone by fine-tuning the pre-trained model (using the skin cancer images) in addition to the pre-trained model (2012 ILSVRC ImageNet dataset [67]). We performed a five-fold cross-validation on the 206 images of the University of Waterloo skin cancer database (four folds with 41 images and the fifth fold with 42 images). Four folds were used to train (fine-tune or train from scratch) the U-Net and the DTP-Net model, and one fold was used for testing. This process was repeated five times, with each fold being used as the test set once. The DSI, MCC, and PSNR scores were then averaged over all five iterations, resulting in the final performance scores. We randomly selected five images from the training fold to set the parameters of the proposed two-stage thresholding technique. Limit1, Limit2, C1, and C2 were heuristically determined as 4, 4, 12, and 12 for the skin cancer dataset. Table 1 presents the DSI, MCC, and PSNR scores of the state-of-the-art thresholding techniques techniques [5,11,12,23,27,28,36,37,38,46,66] and the proposed method. The U-Net model [44] achieved higher performance scores for the skin cancer image dataset. From the results shown in Table 1 and Figure 5, it is evident that the proposed method was quantitatively and qualitatively more accurate in segmenting skin lesion images than the other methods [5,11,12,23,27,28,36,37,38,46] used for comparison.

### 6.2. Experimental Results Using the PCA Board Dataset

This section presents the experimental results of the state-of-the-art thresholding techniques [5,11,12,23,27,28,36,37,38,44,46] using our PCA board database. In order to test the performance of the thresholding techniques under varying conditions, we systematically created the PCA board dataset, including images with different background colors, lighting intensities, and image qualities. The DTP-Net [46] performance was also evaluated by fine-tuning the pre-trained model and training the model from scratch (using the PCA board images) in addition to the pre-trained model provided by the authors of DTP-Net. Similarly, we evaluated U-Net [44] with the Resnet-152 [66] architecture as the backbone by fine-tuning the pre-trained model and training the model from scratch (using the PCA board images) in addition to the pre-trained model (2012 ILSVRC ImageNet dataset [67]). We performed a five-fold cross-validation on the 50 PCA board images (each fold consisting of 10 images). Four folds were used to train (fine-tune or train from scratch) the DTP-Net model, and one fold was used for testing. This process was repeated five times, with each fold being used as the test set once. The DSI, MCC, and PSNR scores were then averaged over all five iterations, resulting in the final performance scores. To evaluate the efficacy of the proposed method for thresholding PCA board images with varying background colors, the parameters of the proposed method were set using five randomly selected green-colored PCA board images from the training fold. Limit1, Limit2, C1, and C2 were heuristically determined to be 2, 4, 6, and 12 for the PCA board dataset. In order to validate the statistical stability of the proposed method, we conducted statistical analyses using the Shapiro–Wilk test [68], a one-way ANOVA [69] and the multiple comparisons test [70] for the proposed method applied to the PCA board dataset. The experimental results of the statistical analysis (presented in Appendix A) provided strong evidence for the robustness and reliability of the proposed method.

**Figure 6:**
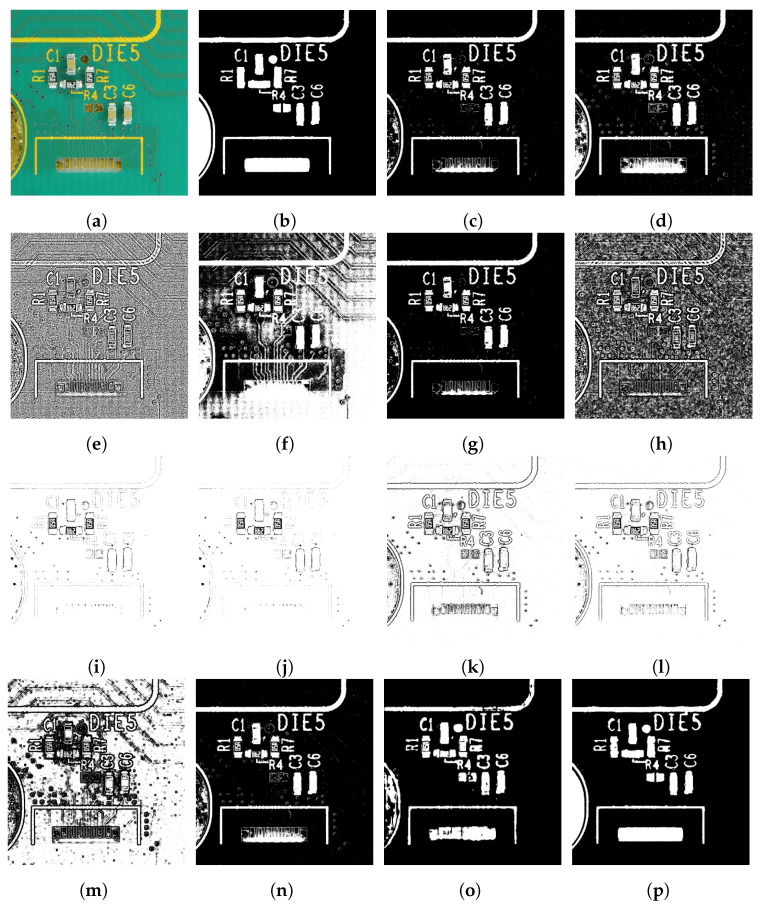
Sample thresholding results using a PCA board image: (**a**) input image, (**b**) ground truth, (**c**) Otsu [11], (**d**) Kapur et al.[12], (**e**) Niblack [23], (**f**) P-tile [27], (**g**) two-peak [27], (**h**) local contrast [27], (**i**) Sauvola et al. [28], (**j**) Wolf and Jolion [36], (**k**) Feng and Tan [37], (**l**) Bradley and Roth [5], (**m**) Singh et al. [38], (**n**) DTP-NET [46] pre-trained model, (**o**) U-Net [44] with Resnet-152 as backbone, (**p**) proposed method.

Table 2 provides the DSI, MCC, and PSNR scores of the thresholding techniques and our proposed method. From the results presented in Table 2 and Figure 6, it is evident that the proposed method achieved more accurate image segmentation results compared to other thresholding techniques in the literature.

**Figure 7:**
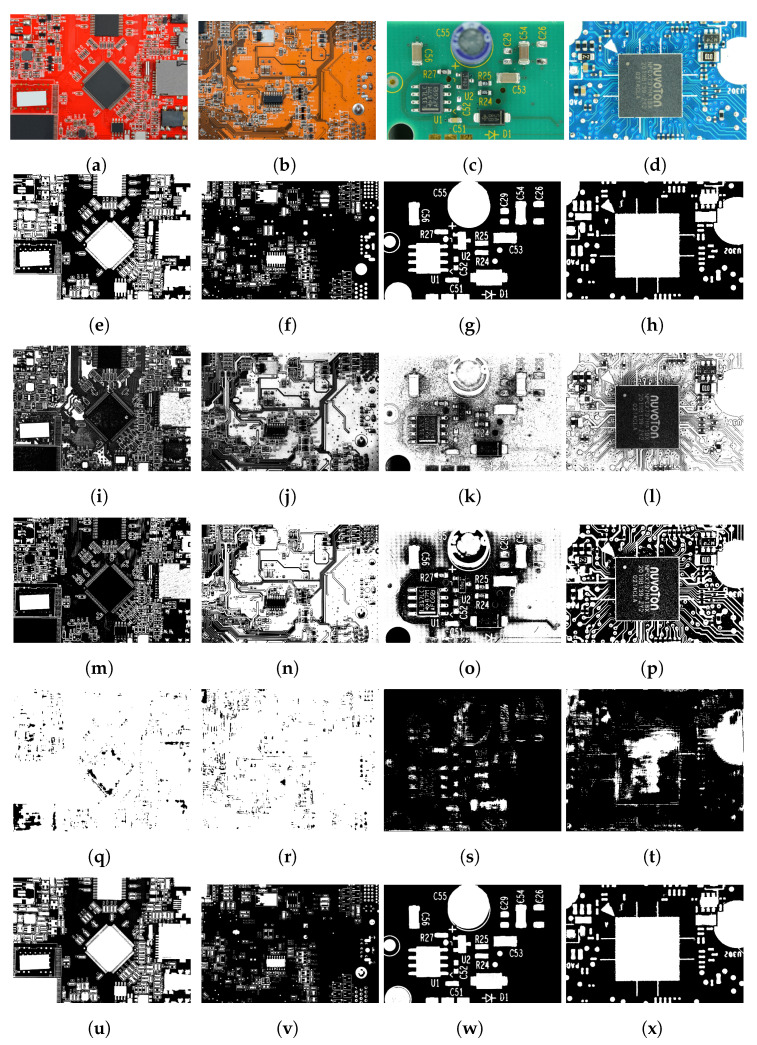
(**a**–**d**) Sample images of the PCA board dataset with varying background colors and (**e**–**h**) corresponding ground truth. (**i**–**l**) Results using Singh et al. [38], (**m**–**p**) DTP-Net fine-tuned model [46], (**q**–**t**) U-Net with Resnet-152 as backbone, and (**u**–**x**) proposed method.

**Figure 8:**
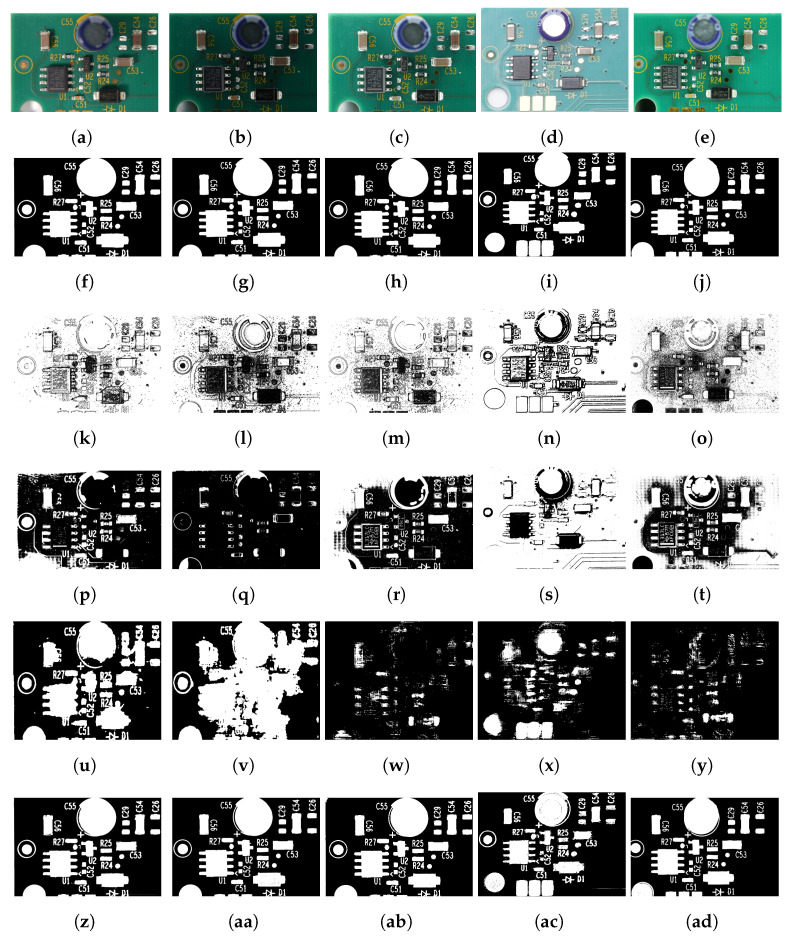
(**a**–**e**) Sample images of the PCA board dataset with varying image intensity and (**f**–**j**) corresponding ground truth. (**k**–**o**) Results using Singh et al. [38], (**p**–**t**) DTP-Net fine-tuned model [46], (**u**–**y**) U-Net (Resnet-152) fine-tuned model, and (**z**–**ad**) proposed method.

To check the effect of background color changes, we deliberately determined the parameters for the algorithm using only green-colored PCA boards (five boards randomly selected from the training folds). Figure 7 depicts the thresholding results for the PCA boards with varying background colors; it is evident that the results of the proposed method were invariant to the changes in the PCA board’s background colors. We also analyzed the performance of the proposed method under varying lighting conditions. From the results illustrated in Figure 8, it is clear that the proposed method was effective in thresholding images with changes in intensity.

**Figure 9:**
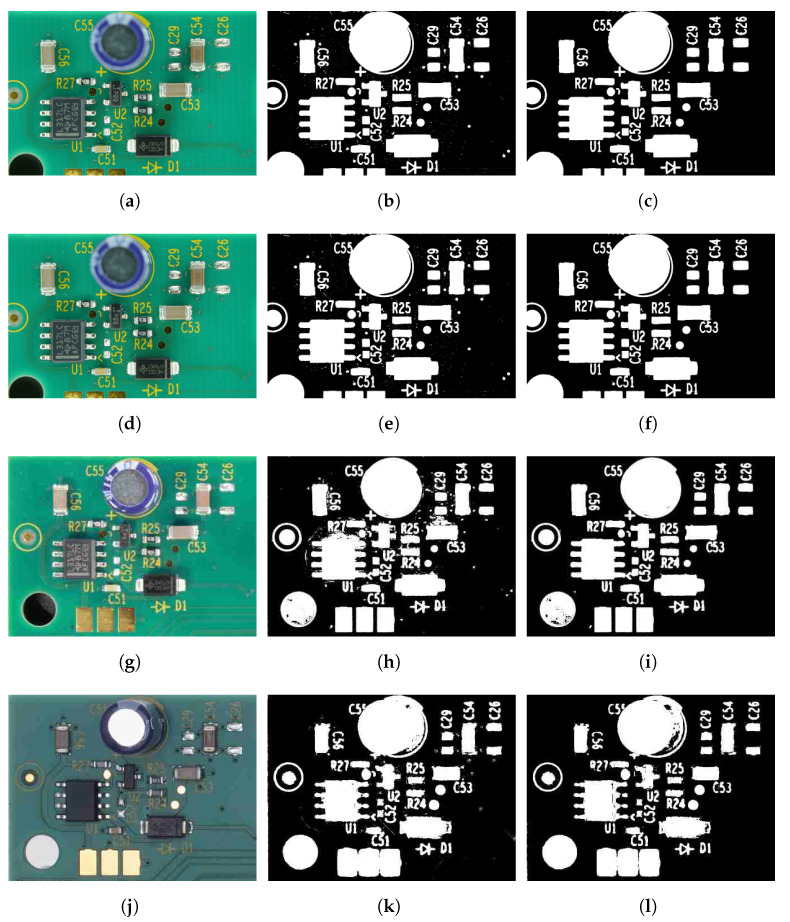
Global and local thresholding results with varying image resolution. Left column (**a**,**d**,**g**,**j**): input PCA board image. Centre column (**b**,**e**,**h**,**k**): global thresholded image. Right column (**c**,**f**,**i**,**l**): local thresholded image. Image resolutions: (**a**) 41.9 MP, (**d**) 10.1 MP, (**g**) 1.9 MP, (**j**) 0.6 MP.

Figure 9 shows the output of the global and local thresholding stages of the proposed two-stage color thresholding technique. We could efficiently suppress the PCA board background without affecting the small foreground objects. Furthermore, the image resolution differed significantly across the sample images, ranging from 0.6 MP to 41.9 MP. The global thresholding stage (center column) effectively suppressed the background colors yet left unwanted traces in the output, such as small particles, shadows, and incorrectly connected components. The local thresholding stage (right column) further improved the results by removing such traces. The overall results showed that the proposed method was invariant to changes in background color, illumination, and image quality.

## 7. Discussion

We proposed a global–local color thresholding technique based on the chromaticity of image pixels. The performance of the proposed method was evaluated using the University of Waterloo skin cancer dataset and a new PCA board dataset. From the experimental results presented in Table 1 and Table 2 and Figure 5 and Figure 6, it is evident that the proposed two-stage global–local color thresholding method outperformed the state-of-the-art thresholding techniques in suppressing the image background.

As depicted in Table 1, the U-Net model achieved the highest performance score (DSI 0.8384, MCC 0.8384, and PSNR 18.79) for the skin cancer image dataset. The proposed method achieved the second highest score (DSI 0.7362, MCC 0.7259, and PSNR 16.2185), and the DTP-Net pre-trained model had the third highest score (DSI 0.6794, MCC 0.6639, and PSNR 15.7098). The U-Net and DTP-Net methods are supervised techniques that require annotated images for training the network. The proposed two-stage color thresholding technique does not require any GT data for training, which is advantageous in the medical domain, as such GT images are limited in number and expensive to obtain.

The PCA board dataset is more complex compared to the skin cancer dataset, since it consists of images depicting small foreground components with varying image quality, intensity, and background color. As presented in Table 2, the proposed method outperformed both the deep-learning-based U-Net model and the DTP-Net fine-tuned model in terms of performance scores. The proposed method achieved a DSI of 0.9846, an MCC of 0.9791, and a PSNR of 23.1545, which were significantly higher than the DSI of 0.6922, MCC of 0.5858, and PSNR of 9.555 achieved by the U-Net model and the DSI of 0.6431, MCC of 0.4996, and PSNR of 8.2162 achieved by the DTP-Net fine-tuned model. The pre-trained network (provided by the DTP-Net authors) was fine-tuned with PCA board images. The U-Net was trained from scratch on PCA board images. To train and fine-tune the network, GT information was required, whereas the proposed method’s parameters were set heuristically based on five green-colored PCA board images. The inadequate performance of the deep learning methods for the PCA board images could be ascribed to the absence of a sufficiently large training dataset and the challenge of precisely thresholding small objects, such as the components typically observed on a PCA board. The DCNN-based methods are not well-equipped to handle such cases [56].

Even though the parameters of the proposed method were set for green-colored PCA boards, the proposed method was efficient in thresholding PCA boards with red, blue, and yellow background colors. In contrast, the performance of U-Net was notably worse for PCA board images with red and yellow backgrounds, which could be attributed to the limited number of training images for these colors in the PCA board dataset. It is worth noting that most PCA boards are typically available in green and blue colors, which could explain the lack of training data for yellow or red backgrounds. The thresholding results presented in Figure 7 and Figure 8 indicate that the U-Net and DTP-net models were not robust for thresholding images with varying background colors and intensities. The results in Figure 7, Figure 8 and Figure 9 show that the proposed thresholding method was invariant to changes in the background color, intensity, and image quality. The findings suggest that for images without visible shadows, such as rows 1 and 2 of Figure 9, the global thresholding result was adequate, and for images with shadows, such as row 3 of Figure 9, the performance could be enhanced using the local thresholding stage. The proposed method was adaptive to changes in image background color, illumination, and image-capturing equipment. We did not have to adjust the parameters to achieve optimal thresholding for varying image conditions—the technique is fully automated, on contrast to many other color thresholding techniques in the literature [11,20,33]. The statistical results obtained from the Shapiro–Wilk analysis [68], one-way ANOVA [69], and multiple comparisons test [70] (presented in Appendix A) provided strong evidence for the robustness and reliability of the proposed method. Overall, our approach showed great potential in tackling the difficulties of image binarization in scenarios where there are limited training data, diverse image conditions, and a need to segment small objects.

To summarize, the proposed color thresholding technique is:

- An unsupervised method, as it does not require any ground-truth data;
- A robust method that is invariant to background color variations and changes in intensity;
- A fully automated color thresholding approach, as there is no need to adjust parameters based on varying image conditions;
- Able to automatically detect the block size for the local thresholding stage;
- Effective at suppressing shadow regions;
- Easily adjustable to different image qualities;
- Efficient in suppressing background pixels of images with tiny foreground components;
- Efficient in determining the threshold value for unimodal, bimodal, and multimodal histograms and also for histograms with sharp peaks and elongated shoulders;
- Effective for symmetric, skewed, or uniform histogram analysis.

## 8. Application Areas

Automated skin lesion detection for disease diagnosis can act as an assistive tool for dermatologists to detect malignant lesions. Such a tool could be used to extract features of skin lesions and monitor changes over time. A robust skin lesion segmentation algorithm would form the basis for such a tool.

The dominant trend of outsourcing the PCA board manufacturing process to lower costs [18,71] and the increasing complexity of these boards have exposed the PCA board supply chain to a range of hardware attacks [72]. Scenarios exist whereby during assembly within the supply chain, a board’s functionality could be maliciously altered by adding, substituting, or removing components. Having human experts check boards during or after assembly is time-consuming, expensive, and prone to error. Therefore, robust computer vision techniques are required to analyze boards for unwanted alterations. The application of such techniques is a multistage process, including some pre-processing. As stated, one key pre-processing technique is distinguishing between background and foreground regions in an image (thresholding). Performing this task accurately makes a big difference in the ability to detect anomalies on a PCA board. Even though there are many well-established methods to perform thresholding, most are not fully automatic for varying PCA board conditions. In [18], the user must manually adjust the parameters to optimize results for backgrounds of varying colors, and the method in [71] requires the user to mark the foreground and background region with the help of an expert. These constraints motivated us to propose the two-stage global–local automatic thresholding technique to distinguish between the background of a PCA board and the foreground components mounted thereon.

**Figure 10:**
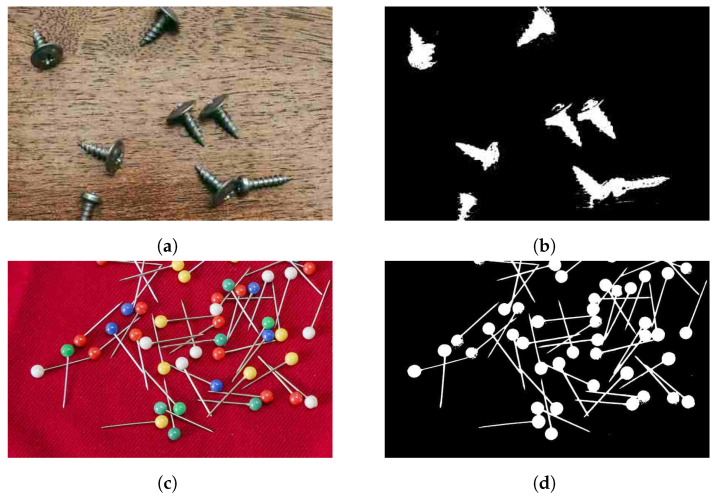

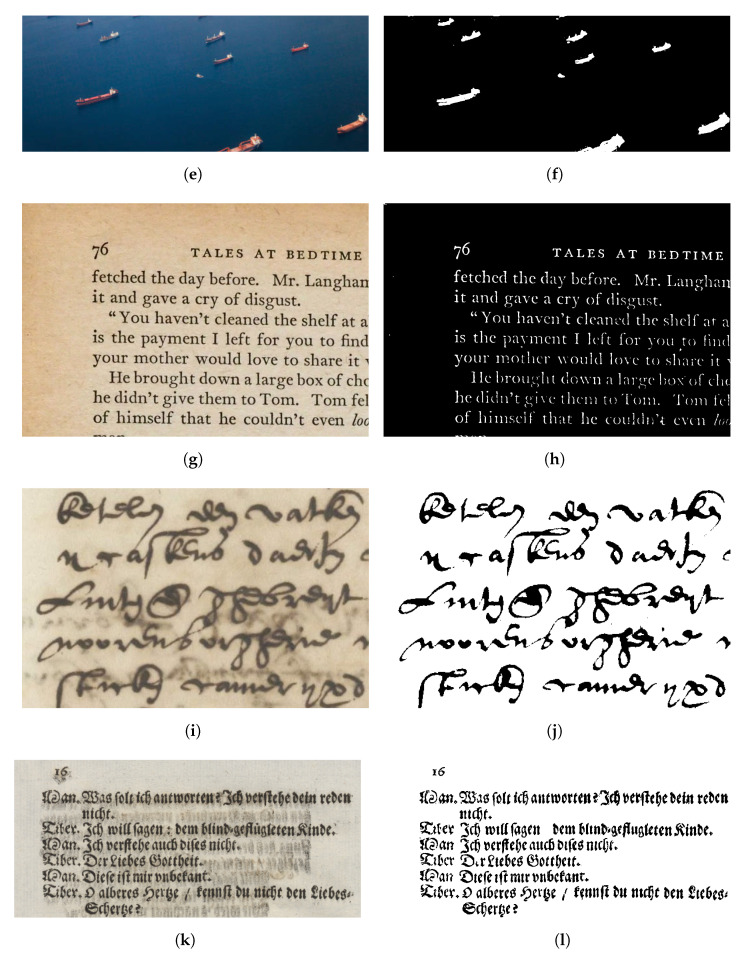
Different sample images and the corresponding globally and locally thresholded outputs: (**a**,**b**) screws on a wooden table; (**c**,**d**) needles on a red background; (**e**,**f**) a drone image of container ships; (**g**,**h**) a photo of an old text document; (**i**–**l**) text with noise in the background from the DIBCO dataset [75].

In addition to the medical image and PCA board analysis, the proposed method could be utilized to analyze a range of images that are relevant for today’s robotic landscape, text detection or recognition, satellite image interpretation, or small object detection tasks. Figure 10 presents some sample images and the corresponding globally and locally thresholded outputs. Moreover, the potential privacy and security aspects of this method have not yet been studied. Due to the automated nature and adaptability of our proposed method, it may act as a building block for systems such as automated web image crawlers and text retrieval systems, which could entail privacy concerns. Existing methods can help to prevent automated text retrieval [73,74].

## 9. Limitations and Future Work

**Figure 11:**
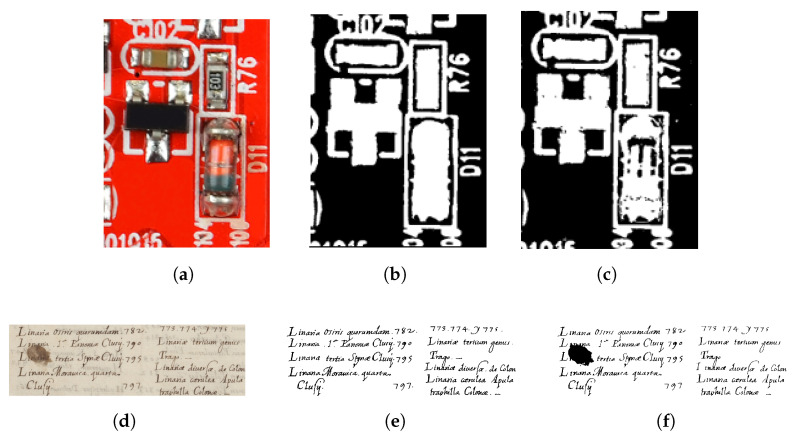
Thresholding results of images with simlarly colored foreground or background regions. Left column: input image. Centre column: ground truth. Right column: image thresholded by the proposed method. The red-colored component D11 in (**a**) is misclassified as the background in (**c**), based on the ground truth (**b**). Ink stains in the input text image (**d**) are misclassified as foreground in (**f**). Images (**d**–**f**) were taken from the DIBCO database [75].

We observed that the presence of any additional, substantially large background region (e.g table or assembly line) affected the determination of background and foreground hues. Hence, the input image must be properly cropped before being passed to the thresholding algorithm. This is not difficult to achieve, since such image backgrounds are uniform and can be cropped automatically. Furthermore, foreground objects that have the same (or a very similar) hue value are classified as background (refer to Figure 11a–c). This is based on the fact that the image segmentation process groups pixels based on a hue range into which foreground objects may fall. Contrarily, background pixels may be classified as foreground if the hue is similar to the foreground color. As the example of the handwritten text document thresholding shown in Figure 11d–f demonstrates, the proposed method in its current format is incapable of suppressing ink stains that resemble the color of the text font. The field of text document binarization has been extensively researched, and there are numerous techniques [7,34,35,75] available in the literature that offer effective solutions for binarizing text images similar to the example shown in Figure 11d. A future research direction may be to incorporate edge information together with chromaticity while determining the foreground or background, which will help to improve the thresholding accuracy of foreground objects with the same color as the background and vice versa. From the experimental results with varying image resolutions (Figure 9), it is evident that the quality of the thresholded output decreased when the image resolution was reduced. Figure 9j (image resolution of 0.6 MP) shows connected blobs in the thresholded output (Figure 9l), compared to the higher-resolution (1.9 MP) image in Figure 9g–i. Some opportunities for future development may include quantitatively measuring the thresholded image quality, so that end users can determine the minimum image resolution that is needed to meet their requirements. U-Net-based segmentation methods have demonstrated impressive performance in many challenging image segmentation tasks [45,76,77], but they require a large amount of annotated training data to achieve such accuracy. Generating ground-truth annotations manually for PCA images using PCA components is a time-consuming and laborious process due to the small size of the components. In the future, if enough annotated data become available, it would be worthwhile to evaluate the performance of U-Net-based segmentation methods for PCA board images.

## 10. Conclusions

In this paper, we presented an unsupervised automatic color thresholding approach to threshold images by isolating significant hue ranges using the image histogram. To evaluate this method, we used a custom-generated PCA board image dataset (with varying background colors, lighting, and image quality) and the University of Waterloo skin cancer image database. We thereby focused on separating the PCA board foreground components from the board’s background and skin lesion binarization. Our proposed global–local color thresholding technique achieved good performance in terms of DSI, MCC, and PSNR scores compared to the naked implementations of state-of-the-art thresholding methods. The proposed method performed well for segmenting lesions from skin cancer images and thresholding small components from PCA board images without any training data. The results showed a clear and robust background–foreground separation across PCA boards with varying background colors, cameras, and lighting setups. With the advancements in PCA board design and components rapidly shrinking in size, such an automated and reliable thresholding method is key for detecting anomalies on PCA boards. The proposed method is fully automatic and does not require any ground-truth information, and it is advantageous in the medical domain, as obtaining ground-truth images is expensive and strenuous. Our approach showed great potential in tackling the difficulties of image binarization in scenarios where there are limited training data, diverse image conditions, and a need to segment small objects.

## Figures and Tables

**Table 1:** DSI, MCC, and PSNR scores for the skin cancer dataset of 206 images used to test various thresholding techniques. The highest performance values are marked with bold font.

Method	DSI	MCC	PSNR
Otsu [11]	0.4242±0.3415	0.4188±0.3447	8.3002±5.7482
Kapur et al. [12]	0.6194±0.2964	0.5950±0.3388	13.8523±6.5068
Niblack [23]	0.1480±0.1098	0.0181±0.0211	3.3668±0.2171
p-tile [27]	0.2614±0.1964	0.2498±0.1753	3.9546±1.0244
Two-peak [27]	0.4505±0.3180	0.4402±0.3437	10.9754±5.8259
Local contrast [27]	0.1652±0.1323	0.0385±0.0536	1.9889±0.5827
Sauvola et al. [28]	0.1812±0.1519	0.2523±0.1659	12.6377±4.6075
Wolf and Jolion [36]	0.2002±0.1396	0.2745±0.1494	12.6661±4.5816
Feng and Tan [37]	0.3254±0.1694	0.3132±0.1700	10.6398±3.2629
Bradley and Roth [5]	0.3000±0.1611	0.3245±0.1639	12.2064±4.0079
Singh et al. [38]	0.3746±0.2612	0.3610±0.2493	6.9653±3.9958
DTP-NET pre-trained model [46]	0.6794±0.2599	0.6639±0.2726	15.7098±5.8357
DTP-NET training from scratch [46]	0.5808±0.2825	0.5695±0.2911	13.3061±5.7834
DTP-NET fine-tuned model [46]	0.6723±0.2616	0.6482±0.2888	15.2232±5.8416
U-Net (Resnet-152) pre-trained [44]	0.0006±0.0035	−0.0336±0.0228	11.2333±3.7380
U-Net (Resnet-152) fine-tuned [44]	0.8384±0.1734	0.8384±0.1651	18.7921±5.0272
Proposed method	0.7362±0.2262	0.7259±0.2334	16.2185±6.3079

**Table 2:** DSI, MCC, and PSNR scores for the PCA board dataset of 50 images used to test various thresholding techniques. The highest performance values are marked with bold font.

Method	DSI	MCC	PSNR
Otsu [11]	0.6621±0.1493	0.6001±0.2161	9.2901±3.678
Kapur et al. [12]	0.6608±0.157	0.5928±0.2039	9.2663±3.5146
Niblack [23]	0.3979±0.1229	0.1068±0.0556	3.2040±0.3981
p-tile [27]	0.4515±0.1462	0.2217±0.1434	3.8677±0.8526
Two-peak [27]	0.4916±0.2491	0.4475±0.2991	7.7011±3.9106
Local contrast [27]	0.4074±0.1081	0.1723±0.0749	4.3730±0.5206
Sauvola et al. [28]	0.3939±0.1393	−0.0601±0.0681	1.5010±0.7247
Wolf and Jolion [36]	0.3927±0.14	−0.0637±0.0647	1.4920±0.7086
Feng and Tan [37]	0.3787±0.1379	−0.0733±0.1184	1.7017±0.8356
Bradley and Roth [5]	0.3881±0.1391	−0.0649±0.0995	1.6008±0.7862
Singh et al. [38]	0.3439±0.1501	−0.0128±0.2084	2.7533±1.3579
DTP-NET pre-trained [46]	0.6040±0.1814	0.4357±0.3327	7.5109±4.5824
DTP-NET training from scratch [46]	0.6197±0.14	0.4597±0.3179	7.7875±4.3566
DTP-NET fine-tuned [46]	0.6431±0.1646	0.4996±0.3178	8.2162±4.2064
U-Net (Resnet-152) pre-trained [44]	0.3207±0.0966	0.0523±0.0712	4.0549±0.8271
U-Net (Resnet-152) training f. s. [44]	0.6922±0.1930	0.5858±0.3065	9.5552±4.5157
U-Net (Resnet-152) fine-tuned [44]	0.6249±0.2790	0.5154±0.3569	9.1995±4.7670
Proposed method	0.9846±0.0149	0.9791±0.0203	23.1545±4.406

**Figure A1:**
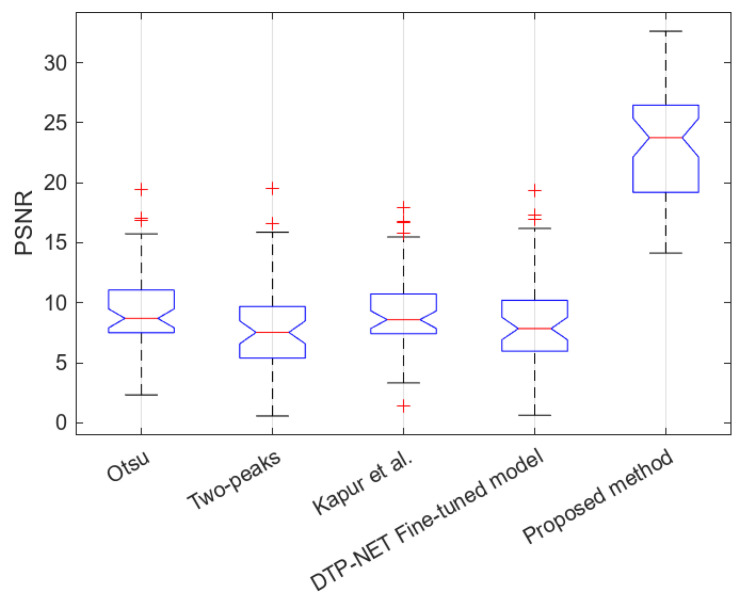
Multiple comparisons test: pair-wise comparison of different methods based on the PSNR scores. The central red line indicates the median PSNR value of each method, and the blue box is the interquartile range (IQR), indicating the 25th and 75th percentiles of the PSNR dataset. The dashed black line represents the whiskers, extending to the minimum and maximum data points. Statistical outliers are marked using the red ’+’ symbol.

**Figure A2:**
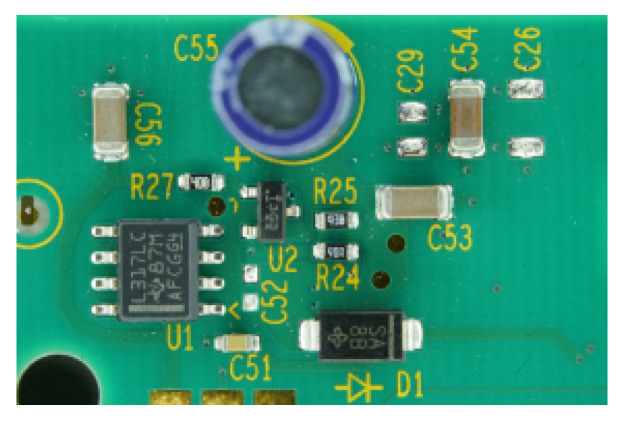
The input image used to test different configurations of the parameters.

## Data Availability

Not applicable.

## Abbreviations

The following abbreviations are used in this manuscript:

CV	Computer vision
DCNN	Deep convolutional neural network
DSI	Dice similarity index
FN	False negative
FP	False positive
GT	Ground truth
HSV	Hue, saturation, value
MCC	Matthews correlation coefficient
MP	Megapixels
PCA	Printed circuit assembly
PCB	Printed circuit board
PMF	Probability mass function
PSNR	Peak signal-to-noise ratio
ROI	Region of interest
TN	True negative
TP	True positive

## Appendix A. Statistical Evaluation of the Proposed Method on the PCA Board Dataset

In order to validate the statistical stability of our algorithm and its performance against competitive methods, we performed a one-way analysis of variance (ANOVA) [69] using the 50 images in the PCA board dataset. In order to apply the ANOVA test, we first performed a Shapiro–Wilk test [68] using the PSNR scores of the top five methods, which are presented in Table 2 (Otsu [11], two-peak [27], Kapur et al. [12], DTP-NET (fine-tuned model) [46], and the proposed method). The Shapiro–Wilk test is a widely used statistical tool for testing the normality assumption of data. The null hypothesis $H_{0}$ is defined as: the sample data (PSNR scores) originate from a normally distributed population. The cut-off significance γ=0.05 was chosen for this analysis. Table A1 shows the *p*-value obtained for the PSNR distribution of each method. The high *p*-values (>γ) in Table A1 indicate that we obtained statistically significant evidence at γ=0.05, showing that there was not enough evidence to reject the null hypothesis, and the sample may be considered normally distributed. The Q–Q plots visually indicate that the PSNR scores were normally distributed, and this backed up the Shapiro–Wilk test results.

**Table A1:** *p*-values obtained for Shapiro–Wilk analysis using the PSNR values of Otsu [11], two-peak [27], Kapur et al. [12], DTP-NET (fine-tuned model) [46], and the proposed method.

	*p*-Value
Otsu [11]	0.1383
Two-peak [27]	0.1404
Kapur et al. [12]	0.0501
DTP-NET fine-tuned model [46]	0.1239
Proposed method	0.4575

After analyzing the normality, we performed a one-way analysis of variance (ANOVA) [69] test with the PSNR scores obtained for Otsu [11], two-peak [27], Kapur et al. [12], DTP-NET (fine-tuned model) [46], and the proposed method. The null hypothesis $H_{0}$ was that the mean of the PSNR scores obtained by the different methods would be equal, and the alternative hypothesis $H_{1}$ was that the mean of the PSNR scores obtained by the different methods would not be equal. A cut-off significance of γ=0.05 was chosen for the analysis. Table A2 shows the *p*-value obtained for the PSNR. The small *p*-value (<γ) indicates that we obtained statistically significant evidence at γ=0.05 to show that there was a difference in the PSNR scores obtained by different methods, so we had sufficient evidence to reject $H_{0}$.

**Table A2:** *p*-value obtained for ANOVA test for Otsu [11], two-peak [27], Kapur et al. [12], DTP-NET (fine-tuned model) [46], and the proposed method.

	PSNR
*p*-value	2.11 × 10^−60^

We also performed a multiple comparisons test [70] to evaluate the pair-wise differences of the PSNR scores obtained from Otsu [11], two-peak [27], Kapur et al. [12], DTP-NET (fine-tuned model) [46], and the proposed method. The null hypothesis $H_{0}$ was that the means of the PSNR scores obtained by the two methods would be equal, and the alternative hypothesis $H_{1}$ was that the means of the PSNR scores obtained by the two methods would not be equal, with a cut-off significance of γ=0.05. Table A3 depicts the results of the pair-wise comparison between the PSNR scores of different methods. The proposed method’s pair-wise comparison results indicated small *p*-values (<γ). Thus, we had statistically significant evidence to reject the null hypothesis, which stated that the mean of the proposed method and the method used for comparison would be equal. It is evident from the graph in Figure A1 that the proposed method achieved better PSNR scores than the other four methods.

**Table A3:** Results of multiple comparisons test: pair-wise comparison of different methods based on the PSNR scores. The third column (Difference) indicates the difference between the means of Method 1 and Method 2. The fourth column (*p*-value) indicates the probability of the means of Method 1 and Method 2 being equal.

Method 1	Method 2	Difference	*p*-Value
Proposed method	Otsu [11]	−13.8644	0.0000
Proposed method	Two-peaks [27]	−15.4534	0.0000
Proposed method	Kapur et al. [12]	−13.8882	0.0000
Proposed method	DTP-NET (fine-tuned model) [46]	−14.9383	0.0000
Otsu [11]	Two-peak [27]	1.5889	0.2719
Otsu [11]	Kapur et al. [12]	0.0237	1.0000
Otsu [11]	DTP-NET (fine-tuned model) [46]	−0.5151	0.6640
Two-peak [27]	Kapur et al. [12]	−1.5652	0.2868
Two-peak [27]	DTP-NET (fine-tuned model) [46]	1.0739	0.9677
Kapur et al. [12]	DTP-NET (fine-tuned model) [46]	1.0502	0.6827

The overall results obtained from the Shapiro–Wilk analysis [68], one-way ANOVA [69], and multiple comparisons test [70] provided strong evidence for the robustness and reliability of the proposed method.

## Appendix B. Configurations of Parameters

In this section, we present some sample experimental results based on different configurations of the parameters Cutoff_Gradient, Cutoff_Area, Window_Size, Limit1, Limit2, C1, and C2 to show how the segmentation quality changed when adjusting the parameters.

As a reminder, a specific hue ‘h’ qualifies as the nominated hue when either the average area around ’h’ (within the Window_Size) is greater than the Cutoff_Area or the average slope surrounding ‘h’ (within the Window_Size) is greater than the Cutoff_Gradient. We set the Cutoff_Gradient value as 0.001 and the optimal Cutoff_Area as 1/180 based on the shape of the hue or saturation histograms. The Window_Size constant was set to 5, which effectively calculated the average gradient and area within a window of five consecutive values. When determining the continuous histogram ranges that qualified as a potential background hue or saturation, we introduced Limit1 (hue continuity) and Limit2 (saturation continuity), which refer to the maximum number of consecutive points on the histogram x-axis that could lie outside of our desired gradient or cumulative area. These parameters provided some flexibility when it came to color discontinuities or variations in the background. Limit1 defines the allowable hue discontinuity, and Limit2 defines the allowable saturation discontinuity. The constants C1 and C2 define the degree of change in local hue and saturation from the global values, respectively. The optimal values for these constants depended on the variance in the image background chromaticity. The example configurations in Table A4 were based on the input image shown in Figure A2.

**Table A4:** Example configurations of parameters.

Thresholding Result	Parameters
	Ideal parameters: determined heuristically. **Cutoff_Gradient**: 0.001 **Cutoff_Area**: 1/180, **Window_Size**: 5 **Limit1**: 4, **Limit2**: 10 **C1**: 5, **C2**: 10
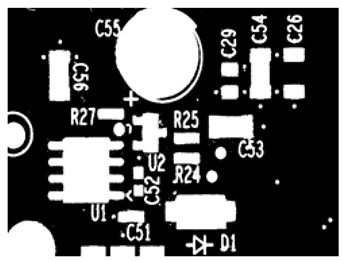
	**Cutoff_Gradient**: 0.1 An increase in **Cutoff_Gradient** by a factor of 100 to 0.1 led to a decrease in the nominated hue or saturation range, which led to the misclassification of some background pixels as foreground.
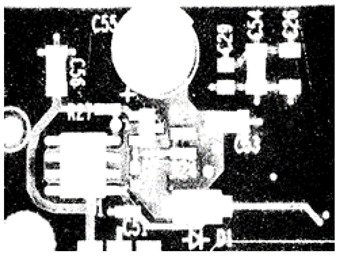
	**Cutoff_Gradient**: 0.00001 A decrease in **Cutoff_Gradient** by a factor of 100 to 0.00001 led to an increase in the nominated hue or saturation range, which led to the misclassification of some foreground pixels as background. **A change in Cutoff_Area led to the same effect.**
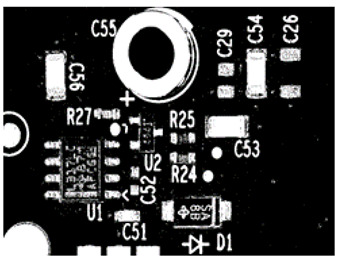
	**Window_Size: 10, Limit1: 8, Limit2: 20** An increase in **Window_Size**, **Limit1**, and **Limit2** by a factor of 2 led to an increase in the nominated hue or saturation range, which led to the misclassification of some foreground pixels as background pixels. **Limit1** defines the allowable hue discontinuity, and **Limit2** defines the allowable saturation discontinuity.
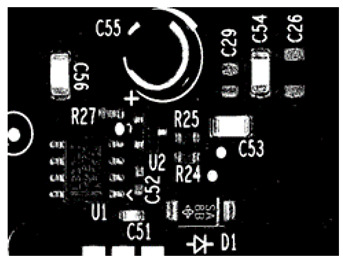
	**Window_Size: 2, Limit1: 2, Limit2: 5** A decrease in **Window_Size**, **Limit1**, and **Limit2** by a factor of 2 led to a decrease in the nominated hue or saturation range, which led to the misclassification of some background pixels as foreground.
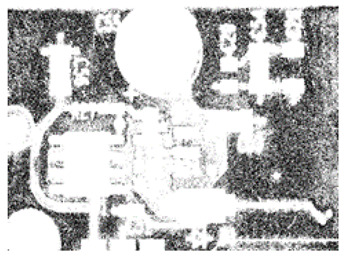
	**C1: 10, C2: 20** An increase in **C1** and **C2** by a factor of 2 led to the misclassification of some foreground pixels as background in the local thresholding stage.
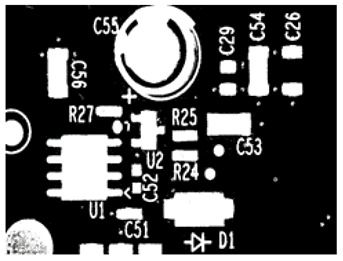
	**C1: 2, C2: 5** A decrease in **C1** and **C2** by a factor of 2 led to the misclassification of background pixels as foreground in the local thresholding stage.

## References

[1] Fan H., Xie F., Li Y., Jiang Z., Liu J. (2017). Automatic segmentation of dermoscopy images using saliency combined with Otsu threshold. Comput. Biol. Med..

[2] Prewitt J.M., Mendelsohn M.L. (1966). The analysis of cell images. Ann. N. Y. Acad. Sci..

[3] Bhandari A., Kumar A., Singh G. (2015). Tsallis entropy based multilevel thresholding for colored satellite image segmentation using evolutionary algorithms. Expert Syst. Appl..

[4] Fan J., Yu J., Fujita G., Onoye T., Wu L., Shirakawa I. (2001). Spatiotemporal segmentation for compact video representation. Signal Process. Image Commun..

[5] Bradley D., Roth G. (2007). Adaptive thresholding using the integral image. J. Graph. Tools.

[6] White J.M., Rohrer G.D. (1983). Image thresholding for optical character recognition and other applications requiring character image extraction. IBM J. Res. Dev..

[7] Shaikh S.H., Maiti A.K., Chaki N. (2013). A new image binarization method using iterative partitioning. Mach. Vis. Appl..

[8] Garcia-Lamont F., Cervantes J., López A., Rodriguez L. (2018). Segmentation of images by color features: A survey. Neurocomputing.

[9] Sahoo P.K., Soltani S.A., Wong A.K. (1988). A survey of thresholding techniques. Comput. Vis. Graph. Image Process..

[10] Hamuda E., Mc Ginley B., Glavin M., Jones E. (2017). Automatic crop detection under field conditions using the HSV colour space and morphological operations. Comput. Electron. Agric..

[11] Otsu N. (1979). A threshold selection method from gray-level histograms. IEEE Trans. Syst. Man Cybern..

[12] Kapur J., Sahoo P., Wong A. (1985). A new method for gray-level picture thresholding using the entropy of the histogram. Comput. Vis. Gr. Image Process.

[13] Pare S., Kumar A., Bajaj V., Singh G.K. (2016). A multilevel color image segmentation technique based on cuckoo search algorithm and energy curve. Appl. Soft Comput..

[14] Mushrif M.M., Ray A.K. (2009). A-IFS histon based multithresholding algorithm for color image segmentation. IEEE Signal Process. Lett..

[15] Cao Z., Zhang X., Mei X. Unsupervised segmentation for color image based on graph theory. Proceedings of the Second International Symposium on Intelligent Information Technology Application.

[16] Harrabi R., Braiek E.B. (2012). Color image segmentation using multi-level thresholding approach and data fusion techniques. EURASIP J. Image Video Process..

[17] Kang S.D., Yoo H.W., Jang D.S. (2007). Color image segmentation based on the normal distribution and the dynamic thresholding. Comput. Sci. Appl. ICCSA.

[18] Mehta D., Lu H., Paradis O.P., MS M.A., Rahman M.T., Iskander Y., Chawla P., Woodard D.L., Tehranipoor M., Asadizanjani N. (2020). The big hack explained: Detection and prevention of PCB supply chain implants. ACM J. Emerg. Technol. Comput. Syst. (JETC).

[19] Mittal H., Pandey A.C., Saraswat M., Kumar S., Pal R., Modwel G. (2021). A comprehensive survey of image segmentation: Clustering methods, performance parameters, and benchmark datasets. Multimed. Tools Appl..

[20] Dirami A., Hammouche K., Diaf M., Siarry P. (2013). Fast multilevel thresholding for image segmentation through a multiphase level set method, Signal Process. Signal Process..

[21] Sezgin M., Sankur B. (2004). Survey over image thresholding techniques and quantitative performance evaluation. J. Electron. Imaging.

[22] Glasbey C.A. (1993). An analysis of histogram-based thresholding algorithms. CVGIP Graph. Model. Image Process..

[23] Niblack W. (1986). An Introduction to Digital Image Processing.

[24] Goh T.Y., Basah S.N., Yazid H., Safar M.J.A., Saad F.S.A. (2018). Performance analysis of image thresholding: Otsu technique. Measurement.

[25] Su H., Zhao D., Elmannai H., Heidari A.A., Bourouis S., Wu Z., Cai Z., Gui W., Chen M. (2022). Multilevel threshold image segmentation for COVID-19 chest radiography: A framework using horizontal and vertical multiverse optimization. Comput. Biol. Med..

[26] Qi A., Zhao D., Yu F., Heidari A.A., Wu Z., Cai Z., Alenezi F., Mansour R.F., Chen H., Chen M. (2023). Directional mutation and crossover boosted ant colony optimization with application to COVID-19 X-ray image segmentation. Comput. Biol. Med..

[27] Parker J.R. (2010). Algorithms for Image Processing and Computer Vision.

[28] Sauvola J., Seppanen T., Haapakoski S., Pietikainen M. Adaptive document binarization. Proceedings of the IEEE Proceedings of the Fourth International Conference on Document Analysis and Recognition, Ulm, Germany, 18–20 August 1997.

[29] Rosenfeld A., Kak A.C. (1982). Digital Picture Processing.

[30] Rosenfeld A., De La Torre P. (1983). Histogram concavity analysis as an aid in threshold selection. IEEE Trans. Syst. Man Cybern..

[31] Mason D., Lauder I., Rutovitz D., Spowart G. (1975). Measurement of C-bands in human chromosomes. Comput. Biol. Med..

[32] Tseng D.C., Li Y.F., Tung C.T. Circular histogram thresholding for color image segmentation. Proceedings of the 3rd International Conference on Document Analysis and Recognition.

[33] Doyle W. (1962). Operations useful for similarity-invariant pattern recognition. J. ACM (JACM).

[34] Pratikakis I., Zagoris K., Barlas G., Gatos B. ICDAR2017 competition on document image binarization (DIBCO 2017). Proceedings of the 2017 14th IAPR International Conference on Document Analysis and Recognition (ICDAR).

[35] Sulaiman A., Omar K., Nasrudin M.F. (2019). Degraded historical document binarization: A review on issues, challenges, techniques, and future directions. J. Imaging.

[36] Wolf C., Jolion J.M. (2004). Extraction and recognition of artificial text in multimedia documents. Form. Pattern Anal. Appl..

[37] Feng M.L., Tan Y.P. (2004). Contrast adaptive binarization of low quality document images. IEICE Electron. Express.

[38] Singh O.I., Sinam T., James O., Singh T.R. (2012). Local contrast and mean thresholding in image binarization. Int. J. Comput. Appl..

[39] Sukesh R., Seuret M., Nicolaou A., Mayr M., Christlein V. (2022). A Fair Evaluation of Various Deep Learning-Based Document Image Binarization Approaches. Document Analysis Systems, Proceedings of the 15th IAPR International Workshop, La Rochelle, France, 22–25 May 2022.

[40] Bankman I. (2008). Handbook of Medical Image PROCESSING and Analysis.

[41] Feng Y., Zhao H., Li X., Zhang X., Li H. (2017). A multi-scale 3D Otsu thresholding algorithm for medical image segmentation. Digit. Signal Process..

[42] Fazilov S.K., Yusupov O.R., Abdiyeva K.S. (2022). Mammography image segmentation in breast cancer identification using the otsu method. Web Sci. Int. Sci. Res. J..

[43] Ramadas M., Abraham A. (2020). Detecting tumours by segmenting MRI images using transformed differential evolution algorithm with Kapur’s thresholding. Neural Comput. Appl..

[44] Ronneberger O., Fischer P., Brox T. (2015). U-Net: Convolutional networks for biomedical image segmentation. Medical Image Computing and Computer-Assisted Intervention, Proceedings of the MICCAI 2015: 18th International Conference, Munich, Germany, 5–9 October 2015.

[45] Punn N.S., Agarwal S. (2022). Modality specific U-Net variants for biomedical image segmentation: A survey. Artif. Intell. Rev..

[46] Venugopal V., Joseph J., Das M.V., Nath M.K. (2022). DTP-Net: A convolutional neural network model to predict threshold for localizing the lesions on dermatological macro-images. Comput. Biol. Med..

[47] Han Q., Wang H., Hou M., Weng T., Pei Y., Li Z., Chen G., Tian Y., Qiu Z. (2023). HWA-SegNet: Multi-channel skin lesion image segmentation network with hierarchical analysis and weight adjustment. Comput. Biol. Med..

[48] Chen S., Zhong L., Qiu C., Zhang Z., Zhang X. (2023). Transformer-based multilevel region and edge aggregation network for magnetic resonance image segmentation. Comput. Biol. Med..

[49] Uslu F., Bharath A.A. (2023). TMS-Net: A segmentation network coupled with a run-time quality control method for robust cardiac image segmentation. Comput. Biol. Med..

[50] Borjigin S., Sahoo P.K. (2019). Color image segmentation based on multi-level Tsallis–Havrda–Charvát entropy and 2D histogram using PSO algorithms. Pattern Recognit..

[51] Fan P., Lang G., Yan B., Lei X., Guo P., Liu Z., Yang F. (2021). A method of segmenting apples based on gray-centered RGB color space. Remote Sens..

[52] Naik M.K., Panda R., Abraham A. (2021). An entropy minimization based multilevel colour thresholding technique for analysis of breast thermograms using equilibrium slime mould algorithm. Appl. Soft Comput..

[53] Ito Y., Premachandra C., Sumathipala S., Premachandra H.W.H., Sudantha B.S. (2021). Tactile paving detection by dynamic thresholding based on HSV space analysis for developing a walking support system. IEEE Access.

[54] Rahimi W.N.S., Ali M.S.A.M. (2020). Ananas comosus crown image thresholding and crop counting using a colour space transformation scheme. Telkomnika.

[55] Minaee S., Boykov Y.Y., Porikli F., Plaza A.J., Kehtarnavaz N., Terzopoulos D. (2021). Image segmentation using deep learning: A survey. IEEE Trans. Pattern Anal. Mach. Intell..

[56] Nguyen N.D., Do T., Ngo T.D., Le D.D. (2020). An evaluation of deep learning methods for small object detection. J. Electr. Comput. Eng..

[57] OpenCV. https://docs.opencv.org/4.x/df/d9d/tutorial_py_colorspaces.html.

[58] Zou K.H., Warfield S.K., Bharatha A., Tempany C.M., Kaus M.R., Haker S.J., Wells W.M., Jolesz F.A., Kikinis R. (2004). Statistical validation of image segmentation quality based on a spatial overlap index1: Scientific reports. Acad. Radiol..

[59] Chicco D., Jurman G. (2020). The advantages of the Matthews correlation coefficient (MCC) over F1 score and accuracy in binary classification evaluation. BMC Genom..

[60] Dhanachandra N., Manglem K., Chanu Y.J. (2015). Image segmentation using K-means clustering algorithm and subtractive clustering algorithm. Procedia Comput. Sci..

[61] University of Waterloo (2022). Vision and Image Processing Lab. Skin Cancer Detection. https://uwaterloo.ca/vision-image-processing-lab/research-demos/skin-cancer-detection.

[62] (2022). Shutterstock. https://www.shutterstock.com.

[63] Giotis I., Molders N., Land S., Biehl M., Jonkman M.F., Petkov N. (2015). MED-NODE: A computer-assisted melanoma diagnosis system using non-dermoscopic images. Expert Syst. Appl..

[64] Yang J., Wu X., Liang J., Sun X., Cheng M.M., Rosin P.L., Wang L. (2019). Self-paced balance learning for clinical skin disease recognition. IEEE Trans. Neural Netw. Learn. Syst..

[65] Lin J., Guo Z., Li D., Hu X., Zhang Y. Automatic classification of clinical skin disease images with additional high-level position information. Proceedings of the 2019 IEEE Chinese Control Conference (CCC).

[66] He K., Zhang X., Ren S., Sun J. Deep residual learning for image recognition. Proceedings of the IEEE Conference on Computer Vision and Pattern Recognition.

[67] Russakovsky O., Deng J., Su H., Krause J., Satheesh S., Ma S., Huang Z., Karpathy A., Khosla A., Bernstein M. (2015). Imagenet large scale visual recognition challenge. Int. J. Comput. Vis..

[68] Shapiro S.S., Wilk M.B. (1965). An analysis of variance test for normality (complete samples). Biometrika.

[69] Girden E.R. (1992). ANOVA: Repeated Measures.

[70] Hochberg Y., Tamhane A.C. (1987). Multiple Comparison Procedures.

[71] Zhao W., Gurudu S.R., Taheri S., Ghosh S., Mallaiyan Sathiaseelan M.A., Asadizanjani N. (2022). PCB Component Detection Using Computer Vision for Hardware Assurance. Big Data Cogn. Comput..

[72] Ghosh S., Basak A., Bhunia S. (2014). How secure are printed circuit boards against trojan attacks?. IEEE Des. Test.

[73] Wu Z., Shen S., Lian X., Su X., Chen E. (2020). A dummy-based user privacy protection approach for text information retrieval. Knowl.-Based Syst..

[74] Wu Z., Shen S., Li H., Zhou H., Lu C. (2021). A basic framework for privacy protection in personalized information retrieval: An effective framework for user privacy protection. J. Organ. End User Comput. (JOEUC).

[75] Mustafa W.A., Khairunizam W., Zunaidi I., Razlan Z.M., Shahriman A.B. (2019). A comprehensive review on document image (DIBCO) database. IOP Conference Series: Materials Science and Engineering.

[76] Zhang S., Zhang C. (2023). Modified U-Net for plant diseased leaf image segmentation. Comput. Electron. Agric..

[77] Zhang Z., Liu Q., Wang Y. (2018). Road extraction by deep residual U-Net. IEEE Geosci. Remote Sens. Lett..

